# Expected values of topological descriptors for possible kink chains of type _2_ ${⊤}_{2}$

**DOI:** 10.3389/fchem.2024.1517892

**Published:** 2025-02-18

**Authors:** Ruxian Chen, Asima Razzaque, Maham Khalil, Salma Kanwal, Saima Noor, Robina Nazir

**Affiliations:** ^1^ School of Public and General Education, Guangzhou Civil Aviation College, Guangzhou, Gaudong, China; ^2^ Institute of Computing Science and Technology, Guangzhou University, Guangzhou, China; ^3^ Department of Basic Science, Preparatory Year, King Faisal University, Al-Ahsa, Saudi Arabia; ^4^ Department of Mathematics, College of Science, King Faisal University, Al-Ahsa, Saudi Arabia; ^5^ Department of Mathematics, Lahore College for Women University, Lahore, Pakistan

**Keywords:** random square-hexagonal kink chains, topological descriptors, expected values, Randic index, Zagreb indices

## Abstract

In this paper, we investigate square-hexagonal chains, a class of systems where the inner dual of a structure with a square-hexagon shape forms a path graph. The specific configuration of square and hexagonal polygons, and how they are concatenated, leads to different types of square-hexagonal chains. A square containing a vertex of degree 2 is classified as having a kink, and the resulting kink is referred to as a type ⊤2 kink. This kink is further subdivided into three types: ⊤12 , _2_ ⊤2 , and _2_ ⊤3 . We focus on the kink chain of type _2_ ⊤2 and compute various topological descriptors for this configuration. By deriving analytical expressions, we determine the maximizing and minimizing values of these descriptors. Additionally, we provide a comprehensive analysis of the expected values for these descriptors and offer a comparison of their behaviors through analytical, numerical, and graphical methods. These results offer insights into the structural properties and behavior of square-hexagonal chains, particularly in relation to the optimization of topological descriptors.

## 1 Preliminaries

Graph theory is a mathematical discipline that studies graphs, which are abstract structures used to model and analyze relationships between objects. A Graph ζ=(V(ζ),E(ζ)) is defined to be the collection of *vertices* (nodes) and *edges* (links or arcs) where V(ζ) and E(ζ) denotes set of vertices and edges respectively. The *order* of a graph, denoted as $n=|\left.V\left(\zeta \right)\right)|$ refers to the total number of vertices in the graph. The *size* of a graph, denoted as m=|E(ζ)| refers to the total number of edges in the graph. The *degree* of a vertex, denoted by deg(w) or $d_{w}$ , is the number of edges connected to that vertex. The distance d(w,x) between two vertices w,x is the length of the shortest path joining them. For basic definitions related to graph theory, we refer (Trinajstic, 1992).

In chemical graph theory, the numerical values assigned to a molecular graph, known as topological indices or molecular descriptors, are often used to correlate with chemical structures and their properties. In other words, topological indices refer to graph invariants or descriptors that have significant chemical relevance. These indices are based on the graphical representation of a molecule and can encode chemical information such as atom types and bond multiplicities. Topological indices are valuable for predicting specific chemical and physical properties of the underlying molecular structure, combining logical and mathematical principles to translate a molecule’s symbolic representation into a useable numerical form. Chemical graph theory, which merges the fields of chemistry and graph theory, uses graphs to represent chemical structures, providing insights into the physical and chemical characteristics of molecules.

The first degree based topological descriptor was introduced by Milan Randic in 1975 in his paper (Randic, 1975) “On characterization of molecular branching.” This index is referred to as Randic index and is defined as

$$R\left(\zeta \right)=\underset{wx\in E\left(\zeta \right)}{\sum }\frac{1}{\sqrt{d_{w}+d_{x}}}.$$

The Randic index has been recognized as a valuable tool in drug design and has been widely used for this purpose in various studies (Randic, 1975).

The first and second Zagreb indices are the oldest degree based graph invariants introduced by Gutman and Trinajstic (Gutman and Trinajstic, 1972b) in 1972. They were later included among topological descriptors and are defined as

$$M_{1}\left(\zeta \right)=\underset{wx\in E\left(\zeta \right)}{\sum }\left(d_{w}+d_{x}\right).$$

$$M_{2}\left(\zeta \right)=\underset{wx\in E\left(\zeta \right)}{\sum }\left(d_{w}\times d_{x}\right).$$

The first and second Zagreb indices were initially applied to branching problem (Gutman et al., 1975b). Later, they found applications in QSPR and QSAR studies (Balaban, 1979; Bonchev and Trinajsti, 2001; Devillers and Balaban, 1999).

The applicability of Zagreb indices motivated the researchers to define different variants of Zagreb indices. The Hyper Zagreb index was put forwarded by Shirdel et al. (Shirdel et al., 2013) in 2013 and is defined as

$$HZ\left(\zeta \right)=\underset{wx\in E\left(\zeta \right)}{\sum }{\left(d_{w}+d_{x}\right)}^{2}.$$

Another variant of Zagreb indices namely, first and second redefined Zagreb indices were introduced by Ranjini et al. (Ranjini et al., 2013)

$$ReZ_{1}\left(\zeta \right)=\underset{wx\in E\left(\zeta \right)}{\sum }\left(\frac{d_{w}+d_{x}}{d_{w}\cdot d_{x}}\right).$$

$$ReZ_{2}\left(\zeta \right)=\underset{wx\in E\left(\zeta \right)}{\sum }\left(\frac{d_{w}\cdot d_{x}}{d_{w}+d_{x}}\right).$$

Motivated by the definitions of first and second Zagreb indices and their chemical applicability, V. Kulli (Kulli, 2017a) introduces the first and second Gourava indices. These topological indices are defined as

$$GO_{1}\left(\zeta \right)=\underset{wx\in E\left(\zeta \right)}{\sum }\left(d_{w}+d_{x}+d_{w}\cdot d_{x}\right).$$

$$GO_{2}\left(\zeta \right)=\underset{wx\in E\left(\zeta \right)}{\sum }\left(d_{w}+d_{x}\right)\left(d_{w}\cdot d_{x}\right).$$

The first and second Revan descriptors were introduced by V. Kulli (Kulli, 2017b) and are defined as

$$R_{1}\left(\zeta \right)=\underset{wx\in E\left(\zeta \right)}{\sum }r_{w}+r_{x}$$

$$R_{2}\left(\zeta \right)=\underset{wx\in E\left(\zeta \right)}{\sum }r_{w}\cdot r_{x}$$

where $r_{w}$ is defined as $r_{w}=\Delta_{\zeta }+\delta_{\zeta }-d_{w}$ , where Δ and δ denotes the maximum and minimum degree among the vertices of ζ .

For more details on the importance of topological indices and their applications see (Noreen and Mahmood, 2018; Wei and Shiu, 2019; Raza, 2020; Wei et al., 2020; Fang et al., 2021; Alraqad et al., 2022; Zhang X. et al., 2023; Zhang Guoping et al., 2023; Hui et al., 2023a; Hui et al., 2023b; Huang et al., 2023). For results related to mathematical properties of the topological indices, we refere (Zhou, 2004; Zhao et al., 2016; Gao et al., 2017; Kulli, 2017c; Kulli, 2017e; Zhang et al., 2024; Govardhan et al., 2024; Prabhu et al., 2024a; Prabhu et al., 2024b).

## 2 Square-hexagonal system and kink chains

A square-hexagonal system, also known as a rectangular hexagonal system, is a connected geometric structure created by joining equal-sized squares and hexagons. This arrangement blends elements of square and hexagonal lattices, forming a distinctive repeating pattern that combines the characteristics of both shapes. The lattice points in this system create a regular and continuous design, where each polygon is linked to its neighbors. Two polygons are considered neighboring if they share a common edge, emphasizing the interconnected nature of this hybrid structure. This system is widely used in crystallography and materials science, particularly for analyzing the structures of materials with hexagonal crystal systems that exhibit square symmetry along specific crystallographic directions. It provides a geometric framework for understanding the arrangement of atoms, ions, or other structural components within such materials.

A square-hexagonal system is a two-dimensional lattice structure that combines square and hexagonal elements in a unified arrangement. In contrast, a square-hexagonal chain is a one-dimensional sequence where square and hexagonal configurations alternate along its length. While both concepts incorporate square and hexagonal features, they differ in their structure and intended applications. The structure of a square-hexagonal chain varies depending on the types of polygons used and how they are concatenated. A square-hexagonal chain composed of n polygons is denoted as $R_{n}$ . If all the polygons in $R_{n}$ are squares, it is referred to as a ***polyomino*** chain (Li et al., 2023). Similarly, if all the polygons are hexagons, $R_{n}$ is called a ***hexagonal chain*** (Alraqad et al., 2022). However, when squares and hexagons alternate in the chain, $R_{n}$ is specifically known as a *phenylene* chain. (as in (Raza, 2021; Shooshtari et al., 2022).

To derive key results, it is important to introduce certain terminologies related to square-hexagonal chains. In graph theory, a kink refers to a point in the graph where there is a sudden change in direction or slope. More precisely, a kink is a vertex whose degree is greater than the degrees of its neighboring vertices, resulting in a bend or angular deviation in the graph’s structure.

Kinks play a significant role in graph analysis, as they often highlight structural changes or key points within the graph. They can influence various graph properties and algorithms, including traversal methods, connectivity analysis, and the identification of critical nodes or hubs in networks. In network analysis, for instance, identifying kinks or high-degree vertices can reveal essential nodes that contribute significantly to the network’s connectivity or exert considerable influence. Moreover, the presence of kinks can affect processes like random walks, as high-degree vertices are more likely to attract repeated visits, thereby altering the overall dynamics of the system.

A polygon at one end of a chain, typically lacking a neighboring polygon on one of its sides, is referred to as a *terminal* polygon. In contrast, a polygon located within the chain, with neighboring polygons on both sides and not positioned at the chain’s ends, is termed a non-terminal polygon.

**FIGURE 1:**
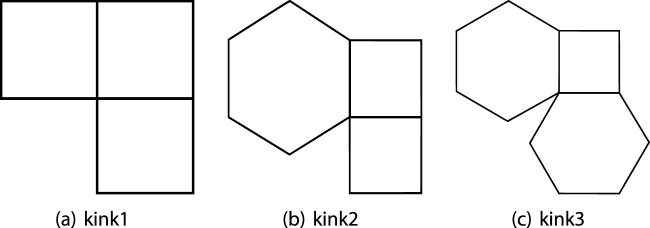
Kinks of Type ⊤12 , ⊤22 and ⊤32 , **(A)** kink1, **(B)** kink2, **(C)** kink3.

If the centers of two adjacent non-terminal polygons are not collinear, the polygon is described as kinked in the chain. There are two types of square-hexagonal kinks, denoted as ⊤1 and ⊤2 (Alraqad et al., 2022). In type ⊤1 , the kink is formed by a hexagon, while in type ⊤2 , square occurs as a kink. A non-terminal hexagon is considered kinked if and only if it contains two consecutive vertices of degree two. Similarly, a non-terminal square is considered kinked if and only if it has a single vertex with a degree of two. Following (Alraqad et al., 2022), we focus on square-hexagonal chains related to the kinks described below:

**(1)** ${Kinks\;}_{1}⊤$ ; A non-terminal hexagon that has exactly two vertices with a degree of two.
**(2)** ${Kinks\;}_{2}{⊤}_{1}$ : A non-terminal square that is adjacent to two squares and has a vertex with a degree of two. (Figure 1A);
**(3)** ${Kinks\;}_{2}{⊤}_{2}$ : A non-terminal square that is adjacent to a square and a hexagon and has a vertex of degree two. (Figure 1B);
**(4)** ${Kinks\;}_{2}{⊤}_{3}$ : A non-terminal square adjacent to two hexagons and has a vertex of degree two. (Figure 1C);

In graph theory, the expected values of topological indices serve as statistical measures of a graph’s structural properties, capturing key characteristics such as connectivity, distances, and vertex degrees. These values are particularly valuable for analyzing and comparing random graphs, optimizing network designs, and predicting behaviors in fields like chemistry, biology, and social networks. Additionally, they enable the development of efficient algorithms for large-scale graph analysis by reducing the computational complexity of calculating indices across various graph models. Our work is motivated from our previous work on the kink chains introduced in (Chunsong et al., 2024). We considered three types of kink chains, ⊤22 , which are categorized as ⊤212 , ⊤222 , and ⊤232 , based on the specific way squares and hexagons are concatenated. For generality, we divided our analysis into two cases: odd and even numbered kink chains, addressing their orders, sizes, and corresponding vertex and edge partitions. Additionally, we calculated the topological indices defined earlier and demonstrated that the second Gourava topological index is a maximizing index, while the redefined first Zagreb index is a minimizing index in both cases. Now, we will determine the expected values of topological descriptors for the newly identified kink chains ⊤212 , ⊤222 , and ⊤232 . These kink chains are defined as;

• **Kink** chains **of**. Type⊤22

**FIGURE 2:**
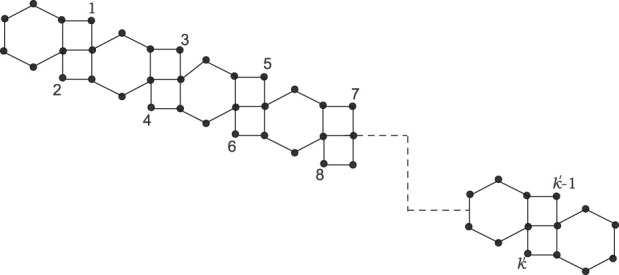
Kinkchain⊤212 .

**FIGURE 3:**
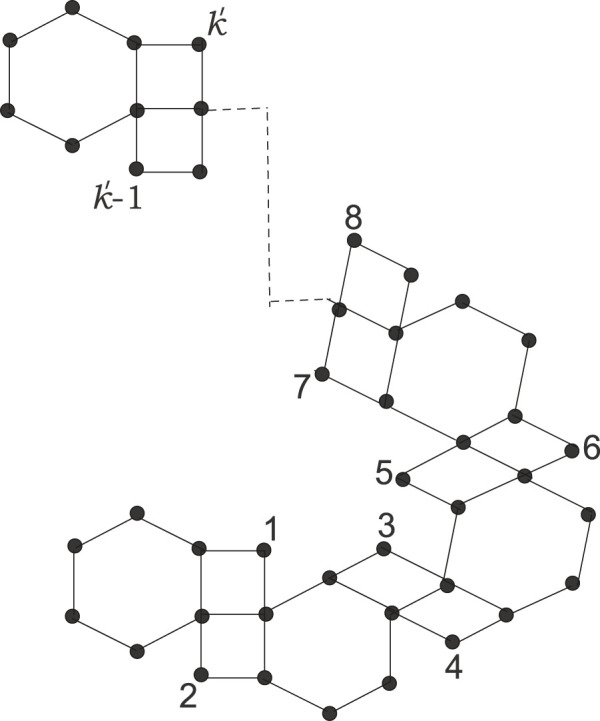
Kinkchain⊤222 .

**FIGURE 4:**
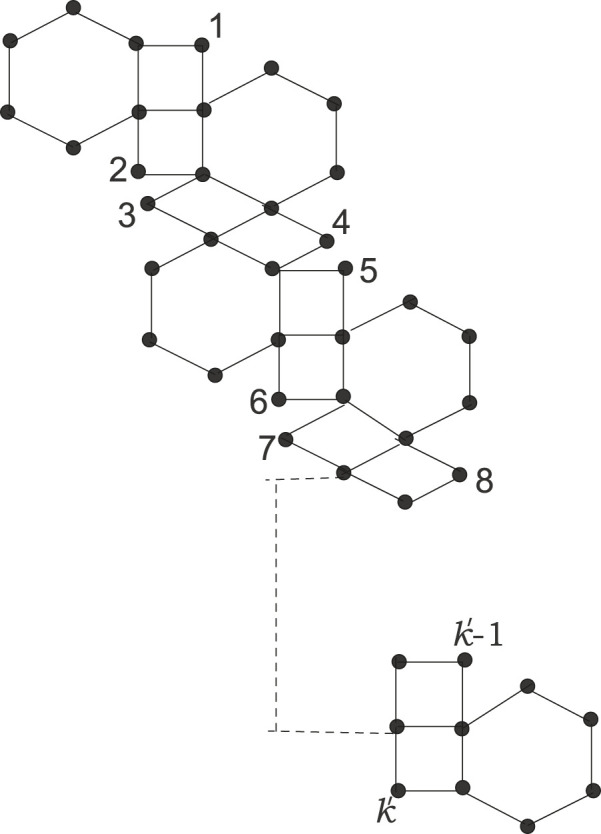
Kinkchain⊤232 .

**(a)** Kinkchain⊤212: A kink chain of type ⊤22 in which no two adjacent vertices in the hexagons have a degree of 2, except at the terminal polygons. It is represented in Figure 2.
**(b)** Kinkchain⊤222: A kink chain of type ⊤22 in which there are exactly two adjacent vertices with a degree of 2 in the hexagons, excluding the terminal polygons. It is represented in Figure 3.
**(c)** Kinkchain⊤232: A kink chain of type ⊤22 in which there are three adjacent vertices with a degree of 2 in the hexagons, excluding the terminal polygons. It is represented in Figure 4.

Observe that there is no edge between two adjacent vertices of degree 2, only one edge between two adjacent vertices of degree 2, and two edges between two adjacent vertices of degree two in kink chains ⊤212 , ⊤222 and ⊤232 respectively, except at terminal polygons. Let $\overset{´}{\kappa }$ represents the number of kinks (squares forming a kink). It is observed that even-numbered kink chains result when two squares are joined at the terminal, while odd-numbered kink chains occur when a hexagon is attached at the terminal. Consequently, an odd-numbered chain corresponds to a square terminal, whereas an even-numbered chain corresponds to a hexagonal terminal. These cases are mathematically represented as $\overset{´}{\kappa }=2n-1$ for odd-numbered chains and $\overset{´}{\kappa }=2n$ for even-numbered chains, where n∈N . The order p of each kink chain is the same across these types and follows the formula $p=6+4\overset{´}{\kappa }$ . However, the size q of the chain differs: for $\overset{´}{\kappa }=2n-1$ (terminal square), $q=\frac{11\overset{´}{\kappa }+13}{2}$ , and for $\overset{´}{\kappa }=2n$ (terminal hexagon), $q=\frac{11\overset{´}{\kappa }+12}{2}$ .

### 2.1 Vertex and corresponding edge partitions of ⊤212 , ⊤222 and ⊤232

Let $E_{ij}=\left\{e=wx;d_{w}=i,d_{x}=j\right\}$ be the subclass of edge sets of ⊤212 , ⊤222 and ⊤232 then $\left|E_{ij}\right|$ depends on number of kinks $\overset{´}{\kappa }$ . Note that there are only (2,2) , (2,3) , (2,4) , (3,4) and (4,4) -type of edges in each kink chain. Table 1 represents the edge partitions of each kink chain accordingly.

**TABLE 1:** Edge partitions of ⊤212 , ⊤222 and ⊤232 ; n∈N .

$\left|E_{ij}\right|$	For $\overset{´}{\kappa }=2n-1$			For $\overset{´}{\kappa }=2n$		
	⊤212	⊤222	⊤232	⊤212	⊤222	⊤232
$\left|E_{22}\right|$	4	$\frac{\overset{´}{\kappa }+7}{2}$	$\overset{´}{\kappa }+3$	6	$\frac{\overset{´}{\kappa }+10}{2}$	$\overset{´}{\kappa }+4$
$\left|E_{23}\right|$	$2\left(\overset{´}{\kappa }+1\right)$	$\frac{3\overset{´}{\kappa }+5}{2}$	4	$2\overset{´}{\kappa }$	$\frac{3\overset{´}{\kappa }+2}{2}$	4
$\left|E_{24}\right|$	$2\overset{´}{\kappa }$	$\frac{3\overset{´}{\kappa }+1}{2}$	$3\overset{´}{\kappa }-1$	$2\overset{´}{\kappa }$	$\frac{3\overset{´}{\kappa }+2}{2}$	$3\overset{´}{\kappa }-2$
$\left|E_{34}\right|$	$\overset{´}{\kappa }+1$	$\frac{3\overset{´}{\kappa }+1}{2}$	2	$\overset{´}{\kappa }$	$\frac{3\overset{´}{\kappa }-2}{2}$	2
$\left|E_{44}\right|$	$\frac{\overset{´}{\kappa }-1}{2}$	$\frac{\overset{´}{\kappa }-1}{2}$	$\frac{3\left(\overset{´}{\kappa }-1\right)}{2}$	$\frac{\overset{´}{\kappa }}{2}$	$\frac{\overset{´}{\kappa }}{2}$	$\frac{3\overset{´}{\kappa }-4}{2}$

Let $V_{i}=\left\{w\in V\left(G\right)|d_{w}=i\right\}$ be the subclass of vertex sets of ⊤212 , ⊤222 and ⊤232 . $\left|V_{i}\right|$ depends on number of kinks $\overset{´}{\kappa }$ . There are only vertices of degree 2, 3 and 4 in each kink chain. Table 2 represents vertex partitions of chains in both cases. Note that the vertex partitions remain same for each kink chain.

**TABLE 2:** Vertex partitions of ⊤212 , ⊤222 and ⊤232 .

$\left|V_{i}\right|$	For $\overset{´}{\kappa }=2n-1$	For $\overset{´}{\kappa }=2n$
	⊤212 , ⊤222 and ⊤232	⊤212 , ⊤222 and ⊤232
$\left|V_{2}\right|$	$2\overset{´}{\kappa }+5$	$2\overset{´}{\kappa }+6$
$\left|V_{3}\right|$	$\overset{´}{\kappa }+1$	$\overset{´}{\kappa }$
$\left|V_{4}\right|$	$\overset{´}{\kappa }$	$\overset{´}{\kappa }$

## 3 Topological descriptors of ⊤212 , ⊤222 and ⊤232

In this section, we will calculate some topological descriptors of ⊤212 , ⊤222 and ⊤232 using Tables 1, 2. Let p denotes the kink chain for kinks of type ⊤22 , where p varies from 1 to 3.

Lemma 1 *For* n∈N *the first Revan index of* Kink chain⊤2p2 *is given as;*

R1⊤2p2=33κ´+49if p=1,2     ; for  κ´=2n−1

R1⊤2p2=33κ´+48if p=1,2     ; for  κ´=2n

R1⊤2p2=32κ´+50          if p=3

Proof. For $\overset{´}{\kappa }=2n-1$

•p=1

R1⊤212=84+72κ´+2+62κ´+5κ´+1+4κ´−12=33κ´+49

•p=2

R1⊤222=8κ´+72+73κ´+52+63κ´+12+53κ´+12+4κ´−12=33κ´+49

•p=3

R1⊤232=8κ´+3+74+63κ´−1+52+43κ´−32=32κ´+50

For $\overset{´}{\kappa }=2n$

•p=1

R1⊤212=86+72κ´+62κ´+5κ´+4κ´2=33κ´+48

•p=2

R1⊤222=8κ´+102+73κ´+22+63κ´+22+53κ´−22+4κ´2=33κ´+48

•p=3

R1⊤232=8κ´+4+74+63κ´−2+52+43κ´−42=32κ´+50

Lemma 2 For n∈N the $2^{nd}$ Revan, $1^{st}$ and $2^{nd}$ Redefined Zagreb, Hyper-Zagreb, $1^{st}$ and $2^{nd}$ Gourava descriptors of kink chains ⊤212 , ⊤222 and ⊤232 are are presented in Table 3;

**TABLE 3:** Topological descriptors of ⊤212 , ⊤222 and ⊤232 ; n∈N .

Topological	For $\overset{´}{\kappa }=2n-1$			For $\overset{´}{\kappa }=2n$		
descriptors	⊤212	⊤222	⊤232	⊤212	⊤222	⊤232
${ℜ}_{2}$	$48\overset{´}{\kappa }+92$	$49\overset{´}{\kappa }+91$	$46\overset{´}{\kappa }+94$	$48\overset{´}{\kappa }+96$	$49\overset{´}{\kappa }+94$	$46\overset{´}{\kappa }+100$
${ReZ}_{1}$	$4\overset{´}{\kappa }+6$	$4\overset{´}{\kappa }+6$	$4\overset{´}{\kappa }+6$	$4\overset{´}{\kappa }+6$	$4\overset{´}{\kappa }+6$	$4\overset{´}{\kappa }+6$
${ReZ}_{2}$	$\frac{817}{105}\overset{´}{\kappa }+\frac{249}{35}$	$\frac{551}{70}\overset{´}{\kappa }+\frac{295}{42}$	$8\overset{´}{\kappa }+\frac{724}{105}$	$\frac{817}{105}\overset{´}{\kappa }+6$	$\frac{551}{70}\overset{´}{\kappa }+\frac{611}{105}$	$8\overset{´}{\kappa }+\frac{584}{105}$
HZ	$203\overset{´}{\kappa }+131$	$205\overset{´}{\kappa }+129$	$220\overset{´}{\kappa }+114$	$203\overset{´}{\kappa }+96$	$205\overset{´}{\kappa }+92$	$220\overset{´}{\kappa }+62$
${GO}_{1}$	$81\overset{´}{\kappa }+61$	$82\overset{´}{\kappa }+60$	$86\overset{´}{\kappa }+56$	$81\overset{´}{\kappa }+48$	$82\overset{´}{\kappa }+46$	$86\overset{´}{\kappa }+38$
${GO}_{2}$	$304\overset{´}{\kappa }+144$	$315\overset{´}{\kappa }+133$	$352\overset{´}{\kappa }+96$	$304\overset{´}{\kappa }+96$	$315\overset{´}{\kappa }+74$	$352\overset{´}{\kappa }$

## 4 Graphical representation of numerical values of topological descriptors of ⊤212 , ⊤222 and ⊤232

**FIGURE 5:**
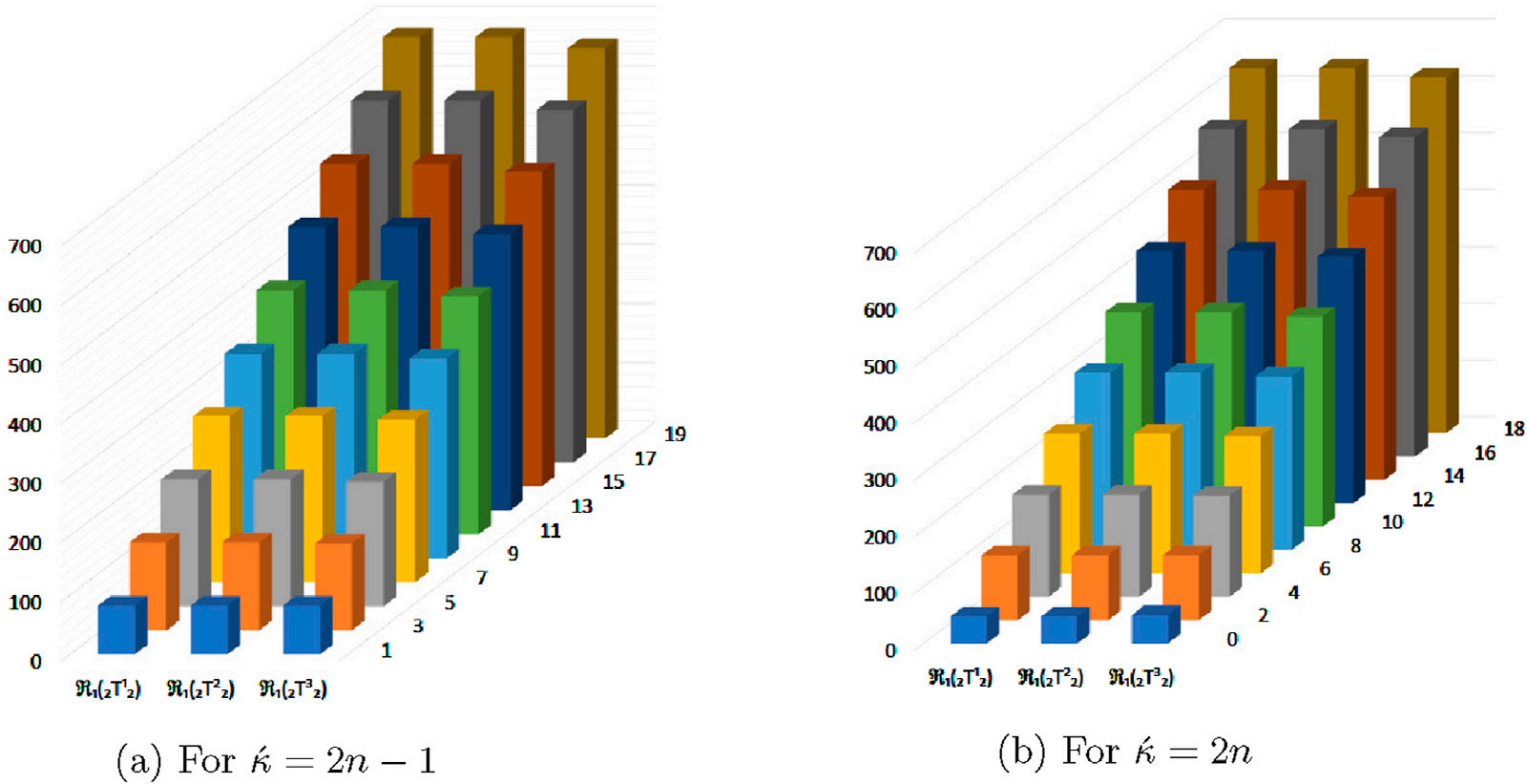
Graphical representation of R1(⊤212) , R1(⊤222) and R1(⊤232) . **(A)** For $\overset{´}{\kappa }=2n-1$ , **(B)** For $\overset{´}{\kappa }=2n$ .

**FIGURE 6:**
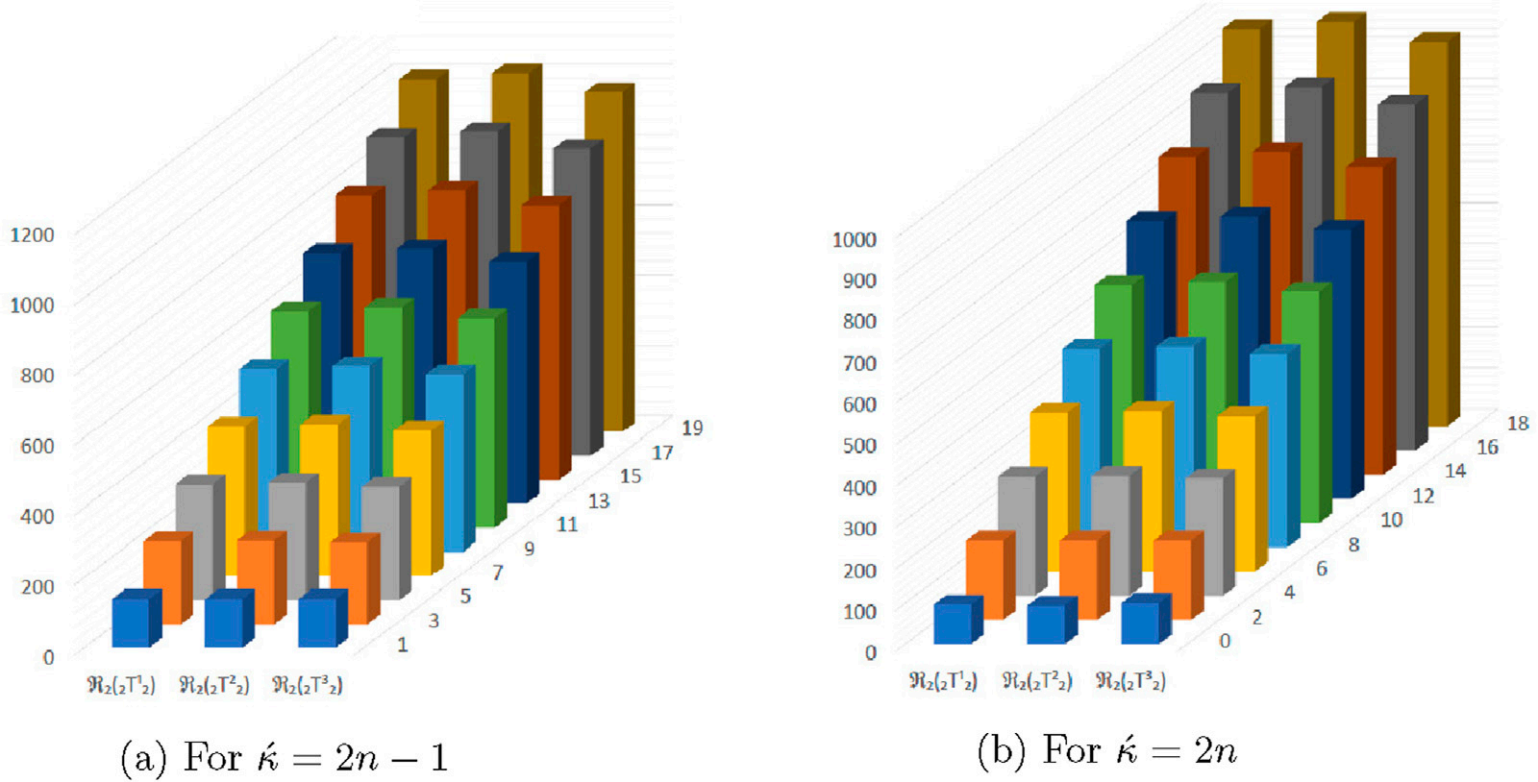
Graphical representation of R2(⊤212) , R2(⊤222) and R2(⊤232) . **(A)** For $\overset{´}{\kappa }=2n-1$ . **(B)** For $\overset{´}{\kappa }=2n$ .

**FIGURE 7:**
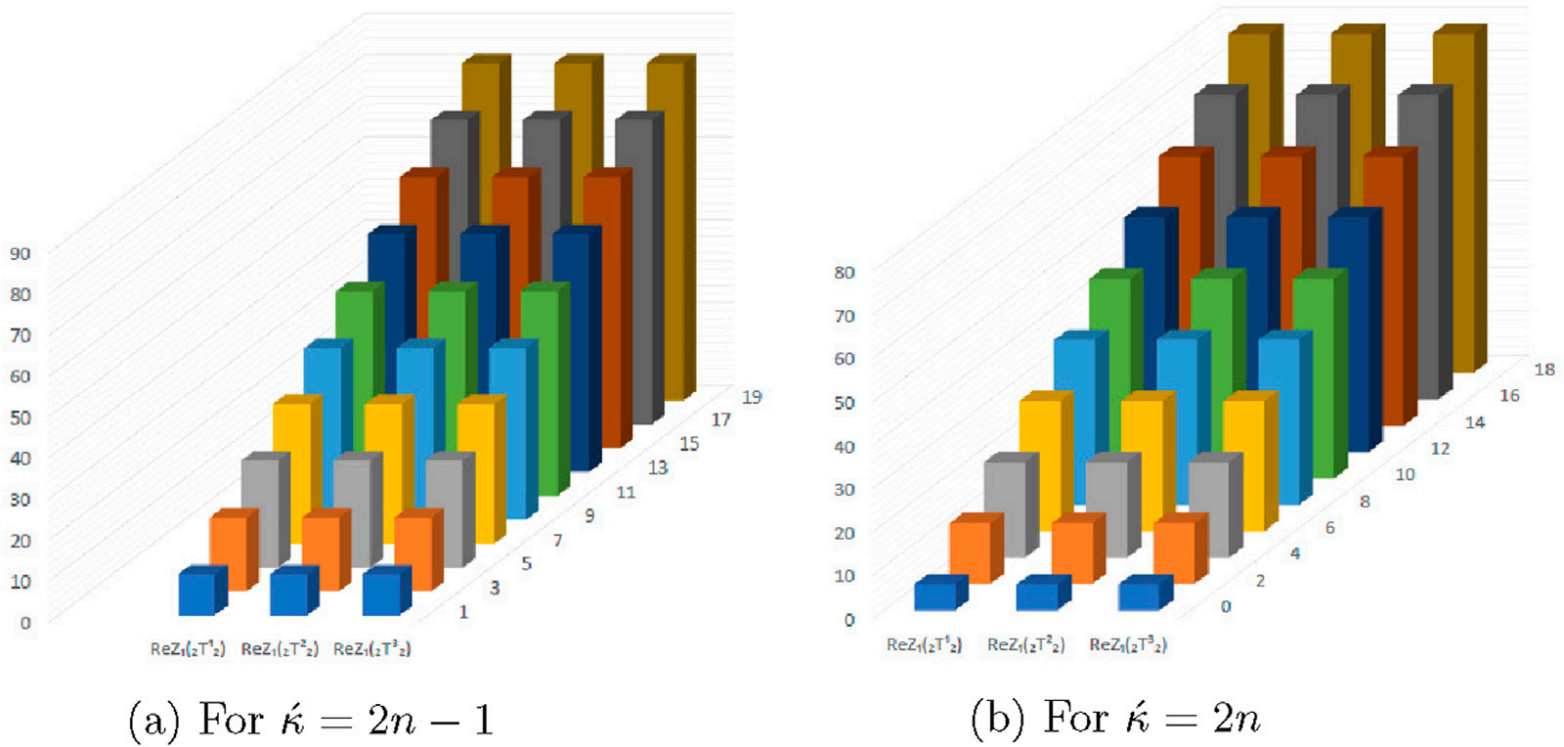
Graphical representation of ReZ1(⊤212) , ReZ1(⊤222) and ReZ1(⊤232) . **(A)** For $\overset{´}{\kappa }=2n-1$ . **(B)** For $\overset{´}{\kappa }=2n$ .

**FIGURE 8:**
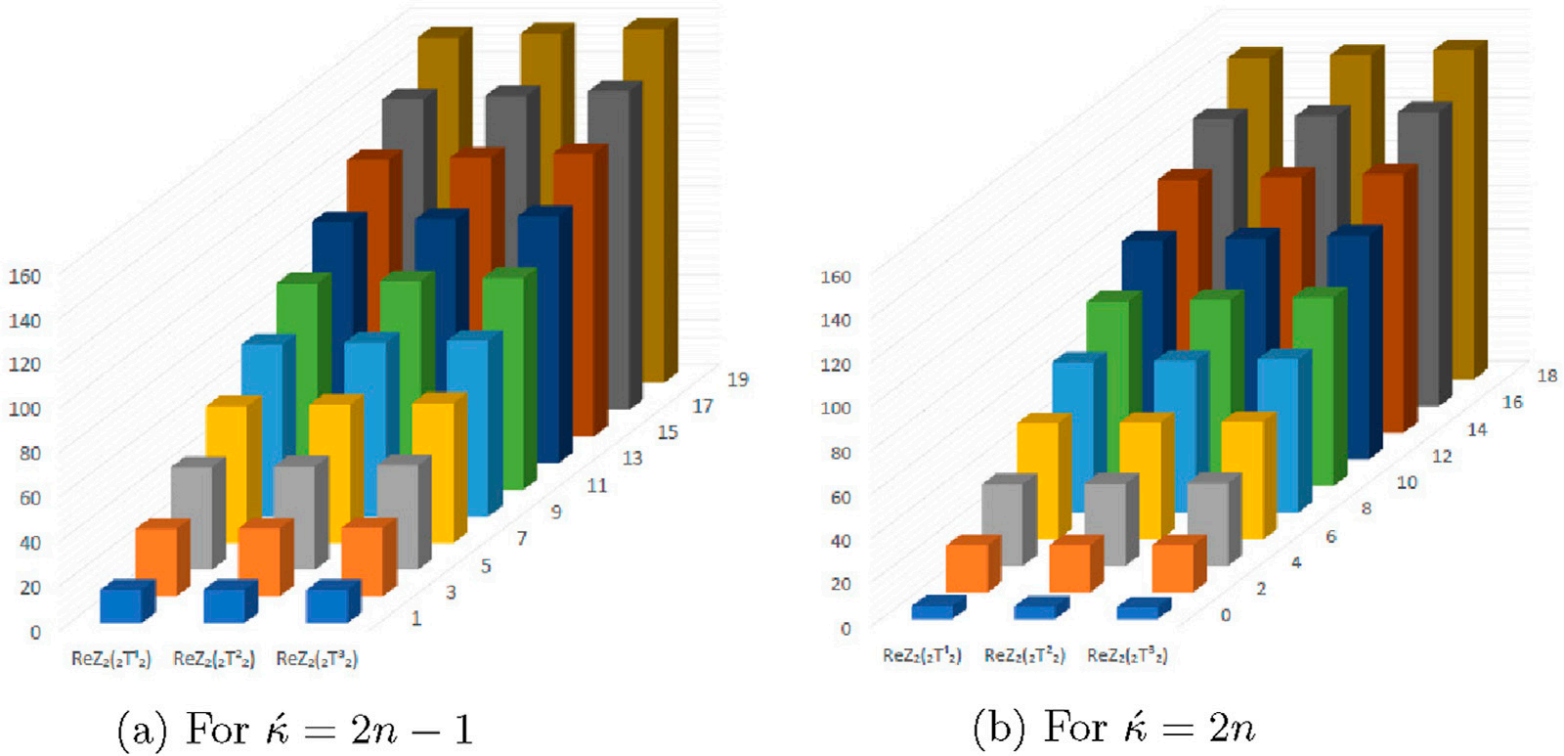
Graphical representation of ReZ2(⊤212) , ReZ2(⊤222) and ReZ2(⊤232) . **(A)** For $\overset{´}{\kappa }=2n-1$ . **(B)** For $\overset{´}{\kappa }=2n$

**FIGURE 9:**
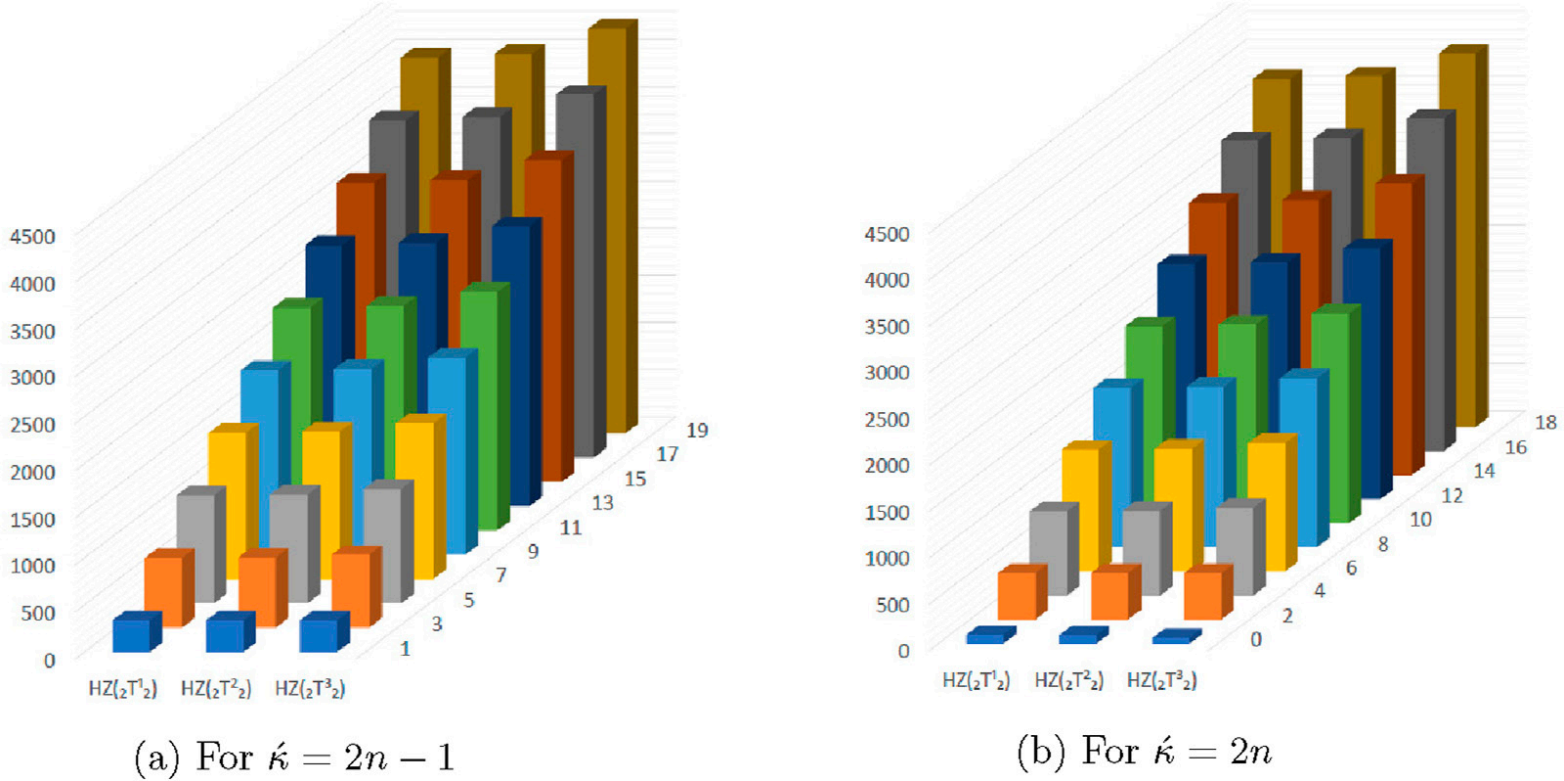
Graphical representation of HZ(⊤212) , HZ(⊤222) and HZ(⊤232) . **(A)** For $\overset{´}{\kappa }=2n-1$ . **(B)** For $\overset{´}{\kappa }=2n$ .

**FIGURE 10:**
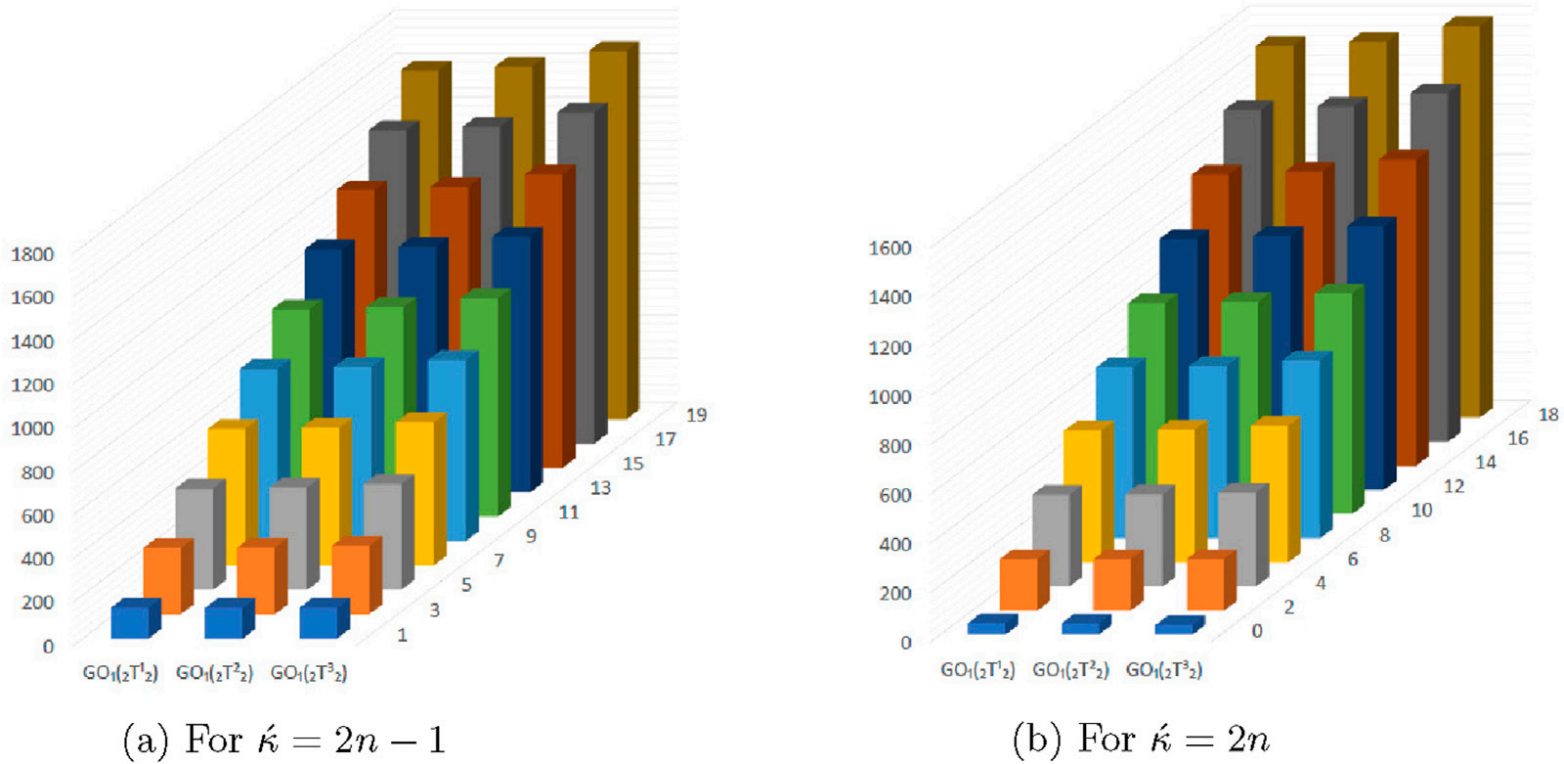
Graphical representation of GO1(⊤212) , GO1(⊤222) and GO1(⊤232) . **(A)** For $\overset{´}{\kappa }=2n-1$ . **(B)** For $\overset{´}{\kappa }=2n$ .

**FIGURE 11:**
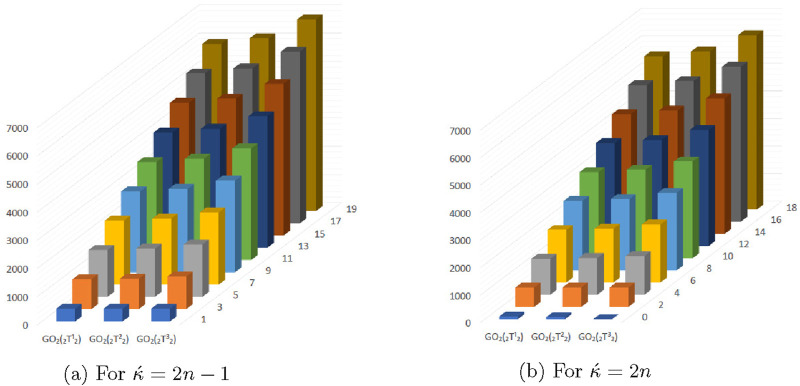
Graphical representation of GO2(⊤212) , GO2(⊤222) and GO2(⊤232) . **(A)** For $\overset{´}{\kappa }=2n-1$ . **(B)** For $\overset{´}{\kappa }=2n$ .

In this section, we compared the above calculated topological descriptors using graphical representation of ⊤212 , ⊤222 and ⊤232 at different values of $\overset{´}{\kappa }$ for odd and even numbered kink chains. From the Figures 5–11 we conclude that $GO_{2}$ descriptor of ⊤212 , ⊤222 and ⊤232 hits a highest value for both $\overset{´}{\kappa }=2n-1$ and $\overset{´}{\kappa }=2n$ . It follows that $GO_{2}$ is a maximizing descriptor. On the other hand $ReZ_{1}$ descriptor of ⊤212 , ⊤222 and ⊤232 reaches a lowest value for both $\overset{´}{\kappa }=2n-1$ and $\overset{´}{\kappa }=2n$ , thus $ReZ_{1}$ is a minimizing descriptor.

## 5 Expected values of topological descriptors of kink chains of type ⊤22

**FIGURE 12:**
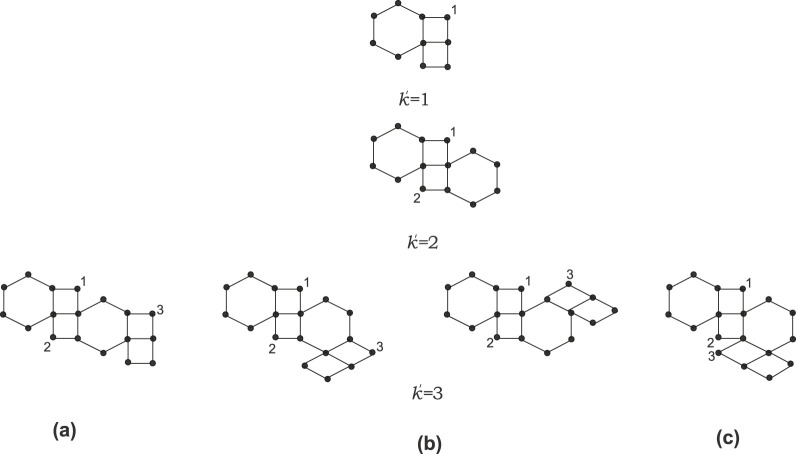
Possible arrangements of kink chains of type ⊤22 .

As we know that there are only three possible kink chains ( ⊤212 , ⊤222 and ⊤232 ) or arrangements of type ⊤22 , holding the conditions to make kink at each step. The kink chains for $\overset{´}{\kappa }=1$ and $\overset{´}{\kappa }=2$ are shown in Figure 12. For $\overset{´}{\kappa }\geq 2$ , terminal polygons are attachable in three different ways, resulting in three types ⊤212 , ⊤222 and ⊤232 . Considering that γ represents the probability of attaching terminal polygons in the first or second kind of arrangement, 1−2γ represents the probability of attaching the terminal polygon in the third type of arrangement. Possible arrangements of kink chains of type ⊤22 are shown in Figure 12.

Let the Kinkchain⊤22 with $\overset{´}{\kappa }$ number of kinks and probability γ is represented by (⊤2γ2)κ´ . We compute *Revan*, *Hyper-Zagreb*, redefined *Zagreb* and *Guorava* descriptors of possible square-hexagonal kink chains (⊤2γ2)κ´ . Let $|E_{ij}|=\beta_{ij}$ denotes the number of edges for (⊤2γ2)κ´ with end vertices of degree *i* and *j* respectively. There are only $\beta_{22}$ , $\beta_{23}$ , $\beta_{24}$ , $\beta_{34}$ and $\beta_{44}$ -type of edges in (⊤2γ2)κ´ . Here Δ⊤22 and δ⊤22 are 4 and 2 implies that (Revan degrees) $r_{s}=4,3,2$ for $d_{s}=2,3$ and 4 respecively. From the definition, the topological descriptors can be expressed as

R1⊤2γ2κ´=8β22⊤2γ2κ´+7β23⊤2γ2κ´+6β24⊤2γ2κ´+5β34⊤2γ2κ´+4β44⊤2γ2κ´

R2⊤2γ2κ´=16β22⊤2γ2κ´+12β23⊤2γ2κ´+8β24⊤2γ2κ´+6β34⊤2γ2κ´+4β44⊤2γ2κ´

ReZ1⊤2γ2κ´=β22⊤2γ2κ´+56β23⊤2γ2κ´+34β24⊤2γ2κ´+712β34⊤2γ2κ´+12β44⊤2γ2κ´

ReZ2⊤2γ2κ´=β22⊤2γ2κ´+65β23⊤2γ2κ´+43β24⊤2γ2κ´+127β34⊤2γ2κ´+2β44⊤2γ2κ´

HZ⊤2γ2κ´=16β22⊤2γ2κ´+25β23⊤2γ2κ´+36β24⊤2γ2κ´+49β34⊤2γ2κ´+64β44⊤2γ2κ´

GO1⊤2γ2κ´=8β22⊤2γ2κ´+11β23⊤2γ2κ´+14β24⊤2γ2κ´+19β34⊤2γ2κ´+24β44⊤2γ2κ´

GO2⊤2γ2κ´=16β22⊤2γ2κ´+30β23⊤2γ2κ´+48β24⊤2γ2κ´+84β34⊤2γ2κ´+128β44⊤2γ2κ´

As (⊤2γ2)κ´ is a random kink chain, it follows R1(⊤2γ2)κ´ , R2(⊤2γ2)κ´ , ReZ1(⊤2γ2)κ´ , ReZ2(⊤2γ2)κ´ , HZ(⊤2γ2)κ´ , GO1(⊤2γ2)κ´ and GO2(⊤2γ2)κ´ are random variables. Let us denote by EnR1=E[R1(⊤2γ2)κ´] , EnR2=E[R2(⊤2γ2)κ´] , EnReZ1=E[ReZ1(⊤2γ2)κ´] , EnReZ2=E[ReZ2(⊤2γ2)κ´] , EnHZ=E[HZ(⊤2γ2)κ´] , EnGO1=E[GO1(⊤2γ2)κ´] and EnGO2=E[GO2(⊤2γ2)κ´] the expected values of these descriptors respectively.

Note that if $\overset{´}{\kappa }$ is odd in kink chains ⊤212 , ⊤222 and ⊤232 , then at ${\left(\overset{´}{\kappa }-1\right)}^{th}$ step, even numbered kink chains are formed, and at ${\left(\overset{´}{\kappa }-2\right)}^{th}$ step, odd numbered kink chains are formed. Similarly, if $\overset{´}{\kappa }$ is even in kink chains ⊤212 , ⊤222 and ⊤232 at ${\left(\overset{´}{\kappa }-1\right)}^{th}$ step, odd numbered kink chains are obtained and at ${\left(\overset{´}{\kappa }-2\right)}^{th}$ step, even numbered kink chains are obtained again. So, we furthur divide our results in two possible stages;

**(**1) At ${\left(\overset{´}{\kappa }-1\right)}^{th}$ stage and (2) At ${\left(\overset{´}{\kappa }-2\right)}^{th}$ stage.

### 5.1 Results at ${\left(\overset{´}{\kappa }-1\right)}^{th}$ stage

The three possible constructions at ${\left(\overset{´}{\kappa }-1\right)}^{th}$ step are as follows:

1.(⊤22)κ´−1→(⊤212)κ´

2.(⊤22)κ´−1→(⊤222)κ´

3.(⊤22)κ´−1→(⊤232)κ´

For $\overset{´}{\kappa }=2n-1$ and $\overset{´}{\kappa }=2n$ ; n∈N the change in edge partitions of (⊤2γ2)κ´ for three possible constructions at ${\left(\overset{´}{\kappa }-1\right)}^{th}$ step are shown in Tables 4, 5 respectively.

**TABLE 4:** Change in edge partitions of (⊤2p2)κ´ at ${\left(k-1\right)}^{th}$ stage for $\overset{´}{\kappa }=2n-1$ ; n∈N .

Type	(⊤22)κ´−1→(⊤2p2)κ´	${\left(\beta_{ij}\right)}_{\overset{´}{\kappa }}={\left(\beta_{ij}\right)}_{\overset{´}{\kappa }-1}\pm$ no. of edges				
p		${\left(\beta_{22}\right)}_{\overset{´}{\kappa }}$	${\left(\beta_{23}\right)}_{\overset{´}{\kappa }}$	${\left(\beta_{24}\right)}_{\overset{´}{\kappa }}$	${\left(\beta_{34}\right)}_{\overset{´}{\kappa }}$	${\left(\beta_{44}\right)}_{\overset{´}{\kappa }}$
1	(⊤22)κ´−1→(⊤212)κ´	${\left(\beta_{22}\right)}_{\overset{´}{\kappa }-1}-2$	${\left(\beta_{23}\right)}_{\overset{´}{\kappa }-1}+4$	${\left(\beta_{24}\right)}_{\overset{´}{\kappa }-1}+2$	${\left(\beta_{34}\right)}_{\overset{´}{\kappa }-1}+2$	${\left(\beta_{44}\right)}_{\overset{´}{\kappa }-1}$
2	(⊤22)κ´−1→(⊤222)κ´	${\left(\beta_{22}\right)}_{\overset{´}{\kappa }-1}-1$	${\left(\beta_{23}\right)}_{\overset{´}{\kappa }-1}+3$	${\left(\beta_{24}\right)}_{\overset{´}{\kappa }-1}+1$	${\left(\beta_{34}\right)}_{\overset{´}{\kappa }-1}+3$	${\left(\beta_{44}\right)}_{\overset{´}{\kappa }-1}$
3	(⊤22)κ´−1→(⊤232)κ´	${\left(\beta_{22}\right)}_{\overset{´}{\kappa }-1}$	${\left(\beta_{23}\right)}_{\overset{´}{\kappa }-1}$	${\left(\beta_{24}\right)}_{\overset{´}{\kappa }-1}+4$	${\left(\beta_{34}\right)}_{\overset{´}{\kappa }-1}$	${\left(\beta_{44}\right)}_{\overset{´}{\kappa }-1}+2$

**TABLE 5:** Change in edge partitions of (⊤2p2)κ´ at ${\left(k-1\right)}^{th}$ stage for $\overset{´}{\kappa }=2n$ ; n∈N .

Type		${\left(\beta_{ij}\right)}_{\overset{´}{\kappa }}={\left(\beta_{ij}\right)}_{\overset{´}{\kappa }-1}\pm$ no. of edges				
		${\left(\beta_{22}\right)}_{\overset{´}{\kappa }}$	${\left(\beta_{23}\right)}_{\overset{´}{\kappa }}$	${\left(\beta_{24}\right)}_{\overset{´}{\kappa }}$	${\left(\beta_{34}\right)}_{\overset{´}{\kappa }}$	${\left(\beta_{44}\right)}_{\overset{´}{\kappa }}$
p=1,2 , 3	(⊤22)κ´−1→(⊤2p2)κ´	${\left(\beta_{22}\right)}_{\overset{´}{\kappa }-1}+2$	${\left(\beta_{23}\right)}_{\overset{´}{\kappa }-1}$	${\left(\beta_{24}\right)}_{\overset{´}{\kappa }-1}+2$	${\left(\beta_{34}\right)}_{\overset{´}{\kappa }-1}$	${\left(\beta_{44}\right)}_{\overset{´}{\kappa }-1}$ +1

Theorem 5.1 Let ⊤22 be a square-hexagonal kink chain and $\overset{´}{\kappa }$ be the number of kinks.

*(* ***a*** *)* For $\overset{´}{\kappa }=2n-1$ ; n∈N

$$E_{n}^{R_{1}}=\overset{´}{\kappa }\left[4\gamma +32\right]-4\gamma +50$$

*(* ***b*** *) For* $\overset{´}{\kappa }=2n$ ; n∈N

$$E_{n}^{R_{1}}=32\overset{´}{\kappa }+50$$

Proof. • (a). When terminal polygon is a square.

For $\overset{´}{\kappa }=1\Rightarrow n=1$ , $E_{1}=82$ , which is indeed true. Let $\overset{´}{\kappa }\geq 2$ , then using Table 4, we get

1. **If** (⊤22)κ´−1→(⊤212)κ´ , then

R1⊤212κ´=8β22⊤22κ´−1−2+7β23⊤22κ´−1+4+6β24⊤22κ´−1+2+5β34⊤22κ´−1+2+4β44⊤22κ´−1

R1⊤212κ´=R1⊤22κ´−1+34 (5.1)

2. **If** (⊤22)κ´−1→(⊤222)κ´ , then

R1⊤222κ´=8β22⊤22κ´−1−1+7β23⊤22κ´−1+3+6β24⊤22κ´−1+1+5β34⊤22κ´−1+3+4β44⊤22κ´−1

R1⊤222κ´=R1⊤22κ´−1+34 (5.2)

3. If (⊤22)κ´−1→(⊤232)κ´ , then

R1⊤232κ´=8β22⊤22κ´−1+7β23⊤22κ´−1+6β24⊤22κ´−1+4+5β34⊤22κ´−1+4β44⊤22κ´−1+2

R1⊤232κ´=R1⊤22κ´−1+32 (5.3)

Thus, we have

Eκ´R1=γR1⊤212κ´+γR1⊤222κ´+1−2γR1⊤232κ´

Using (6.1), (6.2) and (6.3), we get the following relation

Eκ´R1=γR1⊤22κ´−1+34+γR1⊤22κ´−1+34+1−2γR1⊤22κ´−1+32

Eκ´R1=R1⊤22κ´−1+4γ+32

Applying operator E on both sides, we get

$$E\left(E_{\overset{´}{\kappa }}^{R_{1}}\right)=E_{\overset{´}{\kappa }}^{R_{1}}$$

$$E_{\overset{´}{\kappa }}^{R_{1}}=E_{\overset{´}{\kappa }-1}^{R_{1}}+4\gamma +32$$

Using recursive relation upto $\overset{´}{\kappa }-1$ terms

$$\begin{array}{cc} E_{\overset{´}{\kappa }}^{R_{1}} & =E_{\overset{´}{\kappa }-\left(\overset{´}{\kappa }-1\right)}^{R_{1}}+\left(\overset{´}{\kappa }-1\right)\left[4\gamma +32\right]=82+\left(\overset{´}{\kappa }-1\right)\left[4\gamma +32\right]\\ & =\overset{´}{\kappa }\left[4\gamma +32\right]-4\gamma +50 \end{array}$$

• **(b). When terminal polygon is a hexagon.**

For $n=1\Rightarrow \overset{´}{\kappa }=2$ , $E_{2}=114$ , which is indeed true. Let $\overset{´}{\kappa }\geq 2$ , then using Table 5, we get

1. **If** (⊤22)κ´−1→(⊤212)κ´ , then

R1⊤212κ´=8β22⊤22κ´−1+2+7β23⊤22κ´−1+6β24⊤22κ´−1+2+5β34⊤22κ´−1+4β44⊤22κ´−1+1

R1⊤212κ´=R1⊤22κ´−1+32 (5.4)

2. **If** (⊤22)κ´−1→(⊤222)κ´ , then

R1⊤222κ´=8β22⊤22κ´−1+2+7β23⊤22κ´−1+6β24⊤22κ´−1+2+5β34⊤22κ´−1+4β44⊤22κ´−1+1

R1⊤222κ´=R1⊤22κ´−1+32 (5.5)

3. **If** (⊤22)κ´−1→(⊤232)κ´ , then

R1⊤232κ´=6β22⊤22κ´−1+2+7β23⊤22κ´−1+6β24⊤22κ´−1+2+5β34⊤22κ´−1+4β44⊤22κ´−1+1

R1⊤232κ´=R1⊤22κ´−1+32 (5.6)

Thus, we have

Eκ´R1=γR1⊤212κ´+γR1⊤222κ´+1−2γR1⊤232κ´

Using (6.4), (6.5) and (6.6), we get the following relation

Eκ´R1=γR1⊤22κ´−1+32+γR1⊤22κ´−1+32+1−2γR1⊤22κ´−1+32

Eκ´R1=R1⊤22κ´−1+32

Applying operator E on both sides, we get

$$E\left(E_{\overset{´}{\kappa }}^{R_{1}}\right)=E_{\overset{´}{\kappa }}^{R_{1}}$$

$$E_{\overset{´}{\kappa }}^{R_{1}}=E_{\overset{´}{\kappa }-1}^{R_{1}}+32$$

Using recursive relation upto $\overset{´}{\kappa }-2$ terms

$$\begin{array}{cc} E_{\overset{´}{\kappa }}^{R_{1}} & =E_{\overset{´}{\kappa }-\left(\overset{´}{\kappa }-2\right)}^{R_{1}}+\left(\overset{´}{\kappa }-2\right)32=114+\left(\overset{´}{\kappa }-2\right)32\\ & =32\overset{´}{\kappa }+50 \end{array}$$

which completes the proof.

Theorem 5.2 Let ⊤22 be a square-hexagonal kink chain and $\overset{´}{\kappa }$ be the number of kinks. Then

*(* ***a*** *) For* $\overset{´}{\kappa }=2n-1$ ; n∈N

$$E_{\overset{´}{\kappa }}^{R_{2}}=\overset{´}{\kappa }\left[10\gamma +40\right]-10\gamma +100$$

*(* ***b*** *) For* $\overset{´}{\kappa }=2n$ ; n∈N

$$E_{\overset{´}{\kappa }}^{R_{2}}=52\overset{´}{\kappa }+88$$

Proof. • **(a).** For $n=1\Rightarrow \overset{´}{\kappa }=1$ , $E_{1}=140$ , which is indeed true. Let $\overset{´}{\kappa }\geq 2$ , using Table 4, we get

1. **If** (⊤22)n−1→(⊤212)κ´ , then

R2⊤212κ´=16β22⊤22κ´−1−2+12β23⊤22κ´−1+4+8β24⊤22κ´−1+2+6β34⊤22κ´−1+2+4β44⊤22κ´−1

R2⊤212κ´=R2⊤22κ´−1+44 (5.7)

2. **If** (⊤22)κ´−1→(⊤222)κ´ , then

R2⊤222κ´=16β22⊤22κ´−1−1+12β23⊤22κ´−1+3+8β24⊤22κ´−1+1+6β34⊤22κ´−1+3+4β44⊤22κ´−1

R2⊤222κ´=R2⊤22κ´−1+46 (5.8)

3. If (⊤22)κ´−1→(⊤232)κ´ , then

R2⊤232κ´=16β22⊤22κ´−1+12β23⊤22κ´−1+8β24⊤22κ´−1+4+6β34⊤22κ´−1+4β44⊤22κ´−1+2

R2⊤232κ´=R2⊤22κ´−1+40 (5.9)

Thus, we have

Eκ´R2=γR2⊤212κ´+γR2⊤222κ´+1−2γR2⊤232κ´

Using (6.7), (6.8) and (6.9), we get the following relation

Eκ´R2=γR2⊤22κ´−1+44+γR2⊤22κ´−1+46+1−2γR2⊤22κ´−1+40

Eκ´R2=R2⊤22κ´−1+10γ+40

$$E_{\overset{´}{\kappa }}^{R_{2}}=E_{\overset{´}{\kappa }-1}^{R_{2}}+10\gamma +40$$

Using recursive relation upto $\overset{´}{\kappa }-1$ terms

$$\begin{array}{cc} E_{\overset{´}{\kappa }}^{R_{2}} & =E_{\overset{´}{\kappa }-\left(\overset{´}{\kappa }-1\right)}^{R_{2}}+\left(\overset{´}{\kappa }-1\right)\left[10\gamma +40\right]=140+\left(\overset{´}{\kappa }-1\right)\left[10\gamma +40\right]\\ & =\overset{´}{\kappa }\left[10\gamma +40\right]-10\gamma +100 \end{array}$$

• **(b).** For $n=1\Rightarrow \overset{´}{\kappa }=2$ , $E_{2}=192$ , which is indeed true. Let $\overset{´}{\kappa }\geq 2$ , using Table 5, we get

1. **If** (⊤22)κ´−1→(⊤212)κ´ , then

R2⊤212κ´=16β22⊤22κ´−1+2+12β23⊤22κ´−1+8β24⊤22κ´−1+2+6β34⊤22κ´−1+4β44⊤22κ´−1+1

R2⊤212κ´=R1⊤22κ´−1+52 (5.10)

2. **If** (⊤22)κ´−1→(⊤222)κ´ , then

R2⊤222κ´=16β22⊤22κ´−1+2+12β23⊤22κ´−1+8β24⊤22κ´−1+2+6β34⊤22κ´−1+4β44⊤22κ´−1+1

R2⊤222κ´=R2⊤22κ´−1+52 (5.11)

3. If (⊤22)κ´−1→(⊤232)κ´ , then

R2⊤232κ´=16β22⊤22κ´−1+2+12β23⊤22κ´−1+8β24⊤22κ´−1+2+6β34⊤22κ´−1+4β44⊤22κ´−1+1

R2⊤232κ´=R2⊤22κ´−1+52 (5.12)

Thus, we have

Eκ´R2=γR2⊤212κ´+γR2+22⊤22κ´1−2γR2⊤232κ´

Using (6.10), (6.11) and (6.12), we get the following relation

Eκ´R2=γR2⊤22κ´−1+52+γR2⊤22κ´−1+52+1−2γR2⊤22κ´−1+52

Eκ´R2=R2⊤22κ´−1+52

$$E_{\overset{´}{\kappa }}^{R_{2}}=E_{\overset{´}{\kappa }-1}^{R_{2}}+52$$

Using recursive relation upto $\overset{´}{\kappa }-2$ terms

$$\begin{array}{cc} E_{\overset{´}{\kappa }}^{R_{2}} & =E_{\overset{´}{\kappa }-\left(\overset{´}{\kappa }-2\right)}^{R_{2}}+\left(\overset{´}{\kappa }-2\right)52=192+\left(\overset{´}{\kappa }-2\right)52\\ & =52\overset{´}{\kappa }+88 \end{array}$$

which completes the proof.

Theorem 5.3 Let ⊤22 be a square-hexagonal kink chain and $\overset{´}{\kappa }$ be the number of kinks, then

$$E_{\overset{´}{\kappa }}^{ReZ_{1}}=4\overset{´}{\kappa }+6$$

Proof. • **(a).** For $n=1\Rightarrow \overset{´}{\kappa }=1$ , $E_{1}=10$ , which is indeed true. Let $\overset{´}{\kappa }\geq 2$ , using Table 4, we get

1. If (⊤22)κ´−1→(⊤212)κ´ , then

ReZ1⊤212κ´=β22⊤22κ´−1−2+56β23⊤22κ´−1+4+34β24⊤22κ´−1+2+712β34⊤22κ´−1+2+12β44⊤22κ´−1

ReZ1⊤212κ´=ReZ1⊤22κ´−1+4 (5.13)

2. If (⊤22)κ´−1→(⊤222)κ´ , then

ReZ1⊤222κ´=β22⊤22κ´−1−1+56β23⊤22κ´−1+3+34β24⊤22κ´−1+1+712β34⊤22κ´−1+3+12β44⊤22κ´−1

ReZ1⊤222κ´=ReZ1⊤22κ´−1+4 (5.14)

3. If (⊤22)κ´−1→(⊤232)κ´ , then

ReZ1⊤232κ´=β22⊤22κ´−1+56β23⊤22κ´−1+34β24⊤22κ´−1+4+712β34⊤22κ´−1+12β44⊤22κ´−1+2

ReZ1⊤232κ´=ReZ1⊤22κ´−1+4 (5.15)

Thus, we have

Eκ´ReZ1=γReZ1⊤212κ´+γReZ1⊤222κ´+1−2γReZ1⊤232κ´

Using (6.13), (6.14) and (6.15), we get the following relation

Eκ´ReZ1=γReZ1⊤22κ´−1+4+γReZ1⊤22κ´−1+4+1−2γReZ1⊤22κ´−1+4

Eκ´ReZ1=ReZ1⊤22κ´−1+4

Applying operator E on both sides and $∴E\left(E_{\overset{´}{\kappa }}^{ReZ_{1}}\right)=E_{\overset{´}{\kappa }}^{ReZ_{1}}$

$$E_{\overset{´}{\kappa }}^{ReZ_{1}}=E_{\overset{´}{\kappa }-1}^{ReZ_{1}}+4$$

Using recursive relation upto $\overset{´}{\kappa }-1$ terms

$$\begin{array}{cc} E_{\overset{´}{\kappa }}^{ReZ_{1}} & =E_{\overset{´}{\kappa }-\left(\overset{´}{\kappa }-1\right)}^{ReZ1}+\left(\overset{´}{\kappa }-1\right)4=10+\left(\overset{´}{\kappa }-1\right)4\\ & =4\overset{´}{\kappa }+6 \end{array}$$

• **(b).** For $n=1\Rightarrow \overset{´}{\kappa }=2$ , $E_{2}=14$ , which is indeed true. Let $\overset{´}{\kappa }\geq 2$ , using Table 5, we get

1. If (⊤22)κ´−1→(⊤212)κ´ , then

ReZ1⊤212κ´=β22⊤22κ´−1+2+56β23⊤22κ´−1+34β24⊤22κ´−1+2+712β34⊤22κ´−1+12β44⊤22κ´−1+1

ReZ1⊤212κ´=ReZ1⊤22κ´−1+4 (5.16)

2. **If** (⊤22)κ´−1→(⊤222)κ´ , then

ReZ1⊤222κ´=β22⊤22κ´−1+56β23⊤22κ´−1+34β24⊤22κ´−1+4+712β34⊤22κ´−1+12β44⊤22κ´−1+2

ReZ1⊤222κ´=ReZ1⊤22κ´−1+4 (5.17)

3. If (⊤22)κ´−1→(⊤232)κ´ , then

ReZ1⊤232κ´=β22⊤22κ´−1+56β23⊤22κ´−1+34β24⊤22κ´−1+4+712β34⊤22κ´−1+12β44⊤22κ´−1+2

ReZ1⊤232κ´=ReZ1⊤22κ´−1+4 (5.18)

Thus, we have

Eκ´ReZ1=γReZ1⊤212κ´+γReZ1⊤222κ´+1−2γReZ1⊤232κ´

Using (6.16), (6.17) and (6.18), we get the following relation

Eκ´ReZ1=γReZ1⊤22κ´−1+4+γReZ1⊤22κ´−1+4+1−2γReZ1⊤22κ´−1+4

Eκ´ReZ1=ReZ1⊤22κ´−1+4

$$E_{\overset{´}{\kappa }}^{ReZ_{1}}=E_{\overset{´}{\kappa }-1}^{ReZ_{1}}+4$$

Using recursive relation upto $\overset{´}{\kappa }-2$ terms

$$\begin{array}{cc} E_{\overset{´}{\kappa }}^{ReZ_{1}} & =E_{\overset{´}{\kappa }-\left(\overset{´}{\kappa }-2\right)}^{ReZ_{1}}+\left(\overset{´}{\kappa }-2\right)\left(4\right)=14+\left(\overset{´}{\kappa }-2\right)4\\ & =4\overset{´}{\kappa }+6 \end{array}$$

which completes the proof.

Theorem 5.4 Let ⊤22 be a square-hexagonal kink chain and $\overset{´}{\kappa }$ be the number of kinks.

*(* ***a*** *)* For $\overset{´}{\kappa }=2n-1$ ; n∈N

$$E_{\overset{´}{\kappa }}^{ReZ_{2}}=\overset{´}{\kappa }\left[\frac{28}{3}-\frac{73}{105}\gamma \right]+\frac{73}{105}\gamma +\frac{584}{105}$$

*(* ***b*** *)* For $\overset{´}{\kappa }=2n$ ; n∈N

$$E_{\overset{´}{\kappa }}^{ReZ_{2}}=\frac{20}{3}\overset{´}{\kappa }+\frac{288}{35}$$

Proof. • **(a).** For $n=1\Rightarrow \overset{´}{\kappa }=1$ , $E_{1}=\frac{1564}{105}$ , which is indeed true. Let $\overset{´}{\kappa }\geq 2$ , using Table 4, we get

1. If (⊤22)κ´−1→(⊤212)κ´ , then

ReZ2⊤212κ´=β22⊤22κ´−1−2+65β23⊤22κ´−1+4+43β24⊤22κ´−1+2+127β34⊤22κ´−1+2+2β44⊤22κ´−1

ReZ2⊤212κ´=ReZ2⊤22κ´−1+934105 (5.19)

2. If (⊤22)κ´−1→(⊤222)κ´ , then

ReZ2⊤222κ´=β22⊤22κ´−1−1+65β23⊤22κ´−1+3+43β24⊤22κ´−1+1+127β34⊤22κ´−1+3+2β44⊤22κ´−1

ReZ2⊤222κ´=ReZ2⊤22κ´−1+953105 (5.20)

3. **If** (⊤22)κ´−1→(⊤232)κ´ , then

ReZ2⊤232κ´=β22⊤22κ´−1+65β23⊤22κ´−1+43β24⊤22κ´−1+4+127β34⊤22κ´−1+2β44⊤22κ´−1+2

ReZ2⊤232κ´=ReZ2⊤22κ´−1+283 (5.21)

Thus, we have

Eκ´ReZ2=γReZ2⊤212κ´+γReZ2⊤222κ´+1−2γReZ2⊤232κ´

Using (6.19), (6.20) and (6.21), we get the following relation

Eκ´ReZ2=γReZ2⊤22κ´−1+934105+γReZ2⊤22κ´−1+953105+1−2γReZ2⊤22κ´−1+283

Eκ´ReZ2=ReZ2⊤22κ´−1−73105γ+283

$$E_{\overset{´}{\kappa }}^{ReZ_{2}}=E_{\overset{´}{\kappa }-1}^{ReZ_{2}}-\frac{73}{105}\gamma +\frac{28}{3}$$

Using recursive relation upto $\overset{´}{\kappa }-1$ terms

$$\begin{array}{cc} E_{\overset{´}{\kappa }}^{ReZ_{2}} & =E_{\overset{´}{\kappa }-\left(\overset{´}{\kappa }-1\right)}^{ReZ_{2}}+\left(\overset{´}{\kappa }-1\right)\left[\frac{28}{3}-\frac{73}{105}\gamma \right]=\frac{1564}{105}+\left(\overset{´}{\kappa }-1\right)\left[\frac{28}{3}-\frac{73}{105}\gamma \right]\\ & =\overset{´}{\kappa }\left[\frac{28}{3}-\frac{73}{105}\gamma \right]+\frac{73}{105}\gamma +\frac{584}{105} \end{array}$$

• **(b).** For $n=1\Rightarrow \overset{´}{\kappa }=2$ , $E_{2}=\frac{2264}{105}$ , which is indeed true. Let $\overset{´}{\kappa }\geq 2$ , using Table 5, we get

1. If (⊤22)κ´−1→(⊤212)κ´ , then

ReZ2⊤212κ´=β22⊤22κ´−1+2+65β23⊤22κ´−1+43β24⊤22κ´−1+2+127β34⊤22κ´−1+2β44⊤22κ´−1+1

ReZ2⊤212κ´=ReZ2⊤22κ´−1+203 (5.22)

2. **If** (⊤22)κ´−1→(⊤222)κ´ , then

ReZ2⊤222κ´=β22⊤22κ´−1+65β23⊤22κ´−1+43β24⊤22κ´−1+4+127β34⊤22κ´−1+2β44⊤22κ´−1+2

ReZ2⊤222κ´=ReZ2⊤22κ´−1+203 (5.23)

3. **If** (⊤22)κ´−1→(⊤232)κ´ , then

ReZ2⊤232κ´=β22⊤22κ´−1+65β23⊤22κ´−1+43β24⊤22κ´−1+4+127β34⊤22κ´−1+2β44⊤22κ´−1+2

ReZ2⊤232κ´=ReZ2⊤22κ´−1+203 (5.24)

Thus, we have

Eκ´ReZ2=γReZ2⊤212κ´+γReZ2⊤222κ´+1−2γReZ2⊤232κ´

Using (6.22), (6.23) and (6.24), we get the following relation

Eκ´ReZ2=γReZ2⊤22κ´−1+203+γReZ2⊤22κ´−1+203+1−2γReZ2⊤22κ´−1+203

Eκ´ReZ2=ReZ2⊤22κ´−1+203

Applying operator E on both sides and $∴E\left(E_{\overset{´}{\kappa }}^{ReZ_{2}}\right)=E_{\overset{´}{\kappa }}^{ReZ_{2}}$

$$E_{\overset{´}{\kappa }}^{ReZ_{2}}=E_{\overset{´}{\kappa }-1}^{ReZ_{2}}+\frac{20}{3}$$

Using recursive relation upto $\overset{´}{\kappa }-2$ terms

$$\begin{array}{cc} E_{\overset{´}{\kappa }}^{ReZ_{2}}=E_{\overset{´}{\kappa }-\left(\overset{´}{\kappa }-2\right)}^{ReZ_{2}}+\left(\overset{´}{\kappa }-2\right)\frac{20}{3}=\frac{2264}{105}+\left(\overset{´}{\kappa }-2\right)\frac{20}{3}\\ & =\frac{20}{3}\overset{´}{\kappa }+\frac{288}{35} \end{array}$$

which completes the proof.

Theorem 5.5 Let ⊤22 be a square-hexagonal kink chain and $\overset{´}{\kappa }$ be the number of kinks.

*(* ***a*** *)* For $\overset{´}{\kappa }=2n-1$ ; n∈N

$$E_{\overset{´}{\kappa }}^{HZ}=\overset{´}{\kappa }\left[272-64\gamma \right]+64\gamma +62$$

*(* ***b*** *)* For $\overset{´}{\kappa }=2n$ ; n∈N

$$E_{\overset{´}{\kappa }}^{HZ}=168\overset{´}{\kappa }+166$$

Proof. • **(a).** For $n=1\Rightarrow \overset{´}{\kappa }=1$ , $E_{1}=334$ , which is indeed true. Let $\overset{´}{\kappa }\geq 2$ , using Table 4, we get

1. If (⊤22)κ´−1→(⊤212)κ´ , then

HZ⊤212κ´=16β22⊤22κ´−1−2+25β23⊤22κ´−1+4+36β24⊤22κ´−1+2+49β34⊤22κ´−1+2+64β44⊤22κ´−1

HZ⊤212κ´=HZ⊤22κ´−1+238 (5.25)

2. If (⊤22)κ´−1→(⊤222)κ´ , then

HZ⊤222κ´=16β22⊤22κ´−1−1+25β23⊤22κ´−1+3+36β24⊤22κ´−1+1+49β34⊤22κ´−1+3+64β44⊤22κ´−1

HZ⊤222κ´=HZ⊤22κ´−1+242 (5.26)

3. **If** (⊤22)κ´−1→(⊤232)κ´ , then

HZ⊤232κ´=16β22⊤22κ´−1+25β23⊤22κ´−1+36β24⊤22κ´−1+4+49β34⊤22κ´−1+64β44⊤22κ´−1+2

HZ⊤232κ´=HZ⊤22κ´−1+272 (5.27)

Thus, we have

Eκ´HZ=γHZ⊤212κ´+γHZ⊤222κ´+1−2γHZ⊤232κ´

Using (6.25), (6.26) and (6.27), we get the following relation

Eκ´HZ=γHZ⊤22κ´−1+238+γHZ⊤22κ´−1+242+1−2γHZ⊤22κ´−1+272

Eκ´HZ=HZ⊤22κ´−1−64γ+272

Applying operator E on both sides and $∴\;E\left(E_{\overset{´}{\kappa }}^{HZ}\right)=E_{\overset{´}{\kappa }}^{HZ}$

$$E_{\overset{´}{\kappa }}^{HZ}=E_{\overset{´}{\kappa }-1}^{HZ}-64\gamma +272$$

Using recursive relation upto $\overset{´}{\kappa }-1$ terms

$$\begin{array}{cc} E_{\overset{´}{\kappa }}^{HZ} & =E_{\overset{´}{\kappa }-\left(\overset{´}{\kappa }-1\right)}^{HZ}+\left(\overset{´}{\kappa }-1\right)\left(272-64\gamma \right)=334+\left(\overset{´}{\kappa }-1\right)\left(272-64\gamma \right)\\ & =\overset{´}{\kappa }\left[272-64\gamma \right]+64\gamma +62 \end{array}$$

• **(b).** For $n=1\Rightarrow \overset{´}{\kappa }=2$ , $E_{2}=502$ , which is indeed true. Let $\overset{´}{\kappa }\geq 2$ , using Table 5, we get

1. **If** (⊤22)κ´−1→(⊤212)κ´ , then

HZ⊤212κ´=16β22⊤22κ´−1+2+25β23⊤22κ´−1+36β24⊤22κ´−1+2+49β34⊤22κ´−1+64β44⊤22κ´−1+1

HZ⊤212κ´=HZ⊤22κ´−1+168 (5.28)

2. **If** (⊤22)κ´−1→(⊤222)κ´ , then

HZ⊤222κ´=16β22⊤22κ´−1+25β23⊤22κ´−1+36β24⊤22κ´−1+4+49β34⊤22κ´−1+64β44⊤22κ´−1+2

HZ⊤222κ´=HZ⊤22κ´−1+168 (5.29)

3. **If** (⊤22)κ´−1→(⊤232)κ´ , then

HZ⊤232κ´=16β22⊤22κ´−1+2+25β23⊤22κ´−1+36β24⊤22κ´−1+2+49β34⊤22κ´−1+64β44⊤22κ´−1+1

HZ⊤232κ´=HZ⊤22κ´−1+168 (5.30)

Thus, we have

Eκ´HZ=γHZ⊤212κ´+γHZ⊤222κ´+1−2γHZ⊤232κ´

Using (6.28), (6.29) and (6.30), we get the following relation

Eκ´HZ=γHZ1⊤22κ´−1+168+γHZ⊤22κ´−1+168+1−2γHZ⊤22κ´−1+168

Eκ´HZ=HZ⊤22κ´−1+168

$$E_{\overset{´}{\kappa }}^{HZ}=E_{\overset{´}{\kappa }-1}^{HZ}+168$$

Using recursive relation upto $\overset{´}{\kappa }-2$ terms

$$\begin{array}{cc} & =E_{\overset{´}{\kappa }-\left(\overset{´}{\kappa }-2\right)}^{HZ}+\left(\overset{´}{\kappa }-2\right)168=502+\left(\overset{´}{\kappa }-2\right)168\\ & =168\overset{´}{\kappa }+166 \end{array}$$

which completes the proof.

Theorem 5.6 Let ⊤22 be a square-hexagonal kink chain and $\overset{´}{\kappa }$ be the number of kinks.

*(* ***a*** *)* For $\overset{´}{\kappa }=2n-1$ ; n∈N

$$E_{\overset{´}{\kappa }}^{GO_{1}}=\overset{´}{\kappa }\left[104-18\gamma \right]+18\gamma +38$$

*(* ***b*** *)* For $\overset{´}{\kappa }=2n$ ; n∈N

$$E_{\overset{´}{\kappa }}^{GO_{1}}=68\overset{´}{\kappa }+74$$

Proof. • **(a).** For $n=1\Rightarrow \overset{´}{\kappa }=1$ , $E_{1}=142$ , which is indeed true. Let $\overset{´}{\kappa }\geq 2$ , using Table 4, we get

1. If (⊤22)κ´−1→(⊤212)κ´ , then

GO1⊤212κ´=8β22⊤22κ´−1−2+11β23⊤22κ´−1+4+14β24⊤22κ´−1+2+19β34⊤22κ´−1+2+24β44⊤22κ´−1

GO1⊤212κ´=GO1⊤22κ´−1+94 (5.31)

2. **If** (⊤22)κ´−1→(⊤222)κ´ , then

GO1⊤222κ´=8β22⊤22κ´−1−1+11β23⊤22κ´−1+3+14β24⊤22κ´−1+1+19β34⊤22κ´−1+3+24β44⊤22κ´−1

GO1⊤222κ´=GO1⊤22κ´−1+96 (5.32)

3. **If** (⊤22)κ´−1→(⊤232)κ´ , then

GO1⊤232κ´=8β22⊤22κ´−1+11β23⊤22κ´−1+14β24⊤22κ´−1+4+19β34⊤22κ´−1+24β44⊤22κ´−1+2

GO1⊤232κ´=GO1⊤22κ´−1+104 (5.33)

Thus, we have

Eκ´GO1=γGO1⊤212κ´+γGO1⊤222κ´+1−2γGO1⊤232κ´

Using (6.31), (6.32) and (6.33), we get the following relation

Eκ´GO1=γGO1⊤22κ´−1+94+γGO1⊤22κ´−1+96+1−2γGO1⊤22κ´−1+104

Eκ´GO1=GO1⊤22κ´−1−18γ+104

Applying operator E on both sides and $∴\;E\left(E_{\overset{´}{\kappa }}^{GO_{1}}\right)=E_{\overset{´}{\kappa }}^{GO_{1}}$

$$E_{\overset{´}{\kappa }}^{GO_{1}}=E_{\overset{´}{\kappa }-1}^{GO1}-18\gamma +104$$

Using recursive relation upto $\overset{´}{\kappa }-1$ terms

$$\begin{array}{cc} E_{\overset{´}{\kappa }}^{GO_{1}} & =E_{\overset{´}{\kappa }-\left(\overset{´}{\kappa }-1\right)}^{GO_{1}}+\left(\overset{´}{\kappa }-1\right)\left[-18\gamma +104\right]=142+\left(\overset{´}{\kappa }-1\right)\left[104-18\gamma \right]\\ & =\overset{´}{\kappa }\left[104-18\gamma \right]+18\gamma +38 \end{array}$$

• **(b).** For $n=1\Rightarrow \overset{´}{\kappa }=2$ , $E_{2}=210$ , which is indeed true. Let $\overset{´}{\kappa }\geq 2$ , using Table 5, we get

1. **If** (⊤22)κ´−1→(⊤212)κ´ , then

GO1⊤212κ´=8β22⊤22κ´−1+2+11β23⊤22κ´−1+14β24⊤22κ´−1+2+19β34⊤22κ´−1+24β44⊤22κ´−1+1

GO1⊤212κ´=GO1⊤22κ´−1+68 (5.34)

2. If (⊤22)κ´−1→(⊤222)κ´ , then

GO1⊤222κ´=8β22⊤22κ´−1+2+11β23⊤22κ´−1+14β24⊤22κ´−1+2+19β34⊤22κ´−1+24β44⊤22κ´−1+1

GO1⊤222κ´=GO1⊤22κ´−1+68 (5.35)

3. **If** (⊤22)κ´−1→(⊤232)κ´ , then

GO1⊤232κ´=8β22⊤22κ´−1+2+11β23⊤22κ´−1+14β24⊤22κ´−1+2+19β34⊤22κ´−1+24β44⊤22κ´−1+1

GO1⊤232κ´=GO1⊤22κ´−1+68 (5.36)

Thus, we have

Eκ´GO1=γGO1⊤212κ´+γGO1⊤222κ´+1−2γGO1⊤232κ´

Using (6.34), (6.35) and (6.36), we get the following relation

Eκ´GO1=γGO1⊤22κ´−1+68+γGO1⊤22κ´−1+68+1−2γGO1⊤22κ´−1+68

Eκ´GO1=GO1⊤22κ´−1+68

$$E_{\overset{´}{\kappa }}^{GO_{1}}=E_{\overset{´}{\kappa }-1}^{GO_{1}}+68$$

Using recursive relation upto $\overset{´}{\kappa }-2$ terms

$$\begin{array}{cc} E_{\overset{´}{\kappa }}^{GO_{1}} & =E_{\overset{´}{\kappa }-\left(\overset{´}{\kappa }-2\right)}^{GO_{1}}+\left(\overset{´}{\kappa }-2\right)\left(68\right)=210+\left(\overset{´}{\kappa }-2\right)68\\ & =68\overset{´}{\kappa }+74 \end{array}$$

which completes the proof.

Theorem 5.7 Let ⊤22 be a square-hexagonal kink chain and $\overset{´}{\kappa }$ be the number of kinks.

*(* ***a*** *)* For $\overset{´}{\kappa }=2n-1$ ; n∈N

$$E_{\overset{´}{\kappa }}^{GO_{2}}=\overset{´}{\kappa }\left[448-170\gamma \right]+170\gamma$$

*(* ***b*** *)* For $\overset{´}{\kappa }=2n$ ; n∈N

$$E_{\overset{´}{\kappa }}^{GO_{2}}=256\overset{´}{\kappa }+192$$

Proof. • **(a).** For $n=1\Rightarrow \overset{´}{\kappa }=1$ , $E_{1}=448$ , which is indeed true. Let $\overset{´}{\kappa }\geq 2$ , using Table 4, we get

1. **If** (⊤22)κ´−1→(⊤212)κ´ , then

GO2⊤212κ´=16β22⊤22κ´−1−2+30β23⊤22κ´−1+4+48β24⊤22κ´−1+2+84β34⊤22κ´−1+2+128β44⊤22κ´−1

GO2⊤212κ´=GO2⊤22κ´−1+352 (5.37)

2. **If** (⊤22)κ´−1→(⊤222)κ´ , then

GO2⊤222κ´=16β22⊤22κ´−1−1+30β23⊤22κ´−1+3+48β24⊤22κ´−1+1+84β34⊤22κ´−1+3+128β44⊤22κ´−1

GO2⊤222κ´=GO2⊤22κ´−1+374 (5.38)

3. **If** (⊤22)κ´−1→(⊤232)κ´ , then

GO2⊤232κ´=16β22⊤22κ´−1+30β23⊤22κ´−1+48β24⊤22κ´−1+4+84β34⊤22κ´−1+128β44⊤22κ´−1+2

GO2⊤232κ´=GO2⊤22κ´−1+448 (5.39)

Thus, we have

Eκ´GO2=γGO2⊤212κ´+γGO2⊤222κ´+1−2γGO2⊤232κ´

Using (6.37), (6.38) and (6.39), we get the following relation

Eκ´GO2=γGO2⊤22κ´−1+352+γGO2⊤22κ´−1+374+1−2γGO2⊤22κ´−1+448

Eκ´GO2=GO2⊤22κ´−1−170γ+448

Applying operator E on both sides and $∴\;E\left(E_{\overset{´}{\kappa }}^{GO_{2}}\right)=E_{\overset{´}{\kappa }}^{GO_{2}}$

$$E_{\overset{´}{\kappa }}^{GO_{2}}=E_{\overset{´}{\kappa }-1}^{GO_{2}}-170\gamma +448$$

Using recursive relation upto $\overset{´}{\kappa }-1$ terms

$$\begin{array}{cc} E_{\overset{´}{\kappa }}^{GO_{2}} & =E_{\overset{´}{\kappa }-\left(\overset{´}{\kappa }-1\right)}^{GO_{2}}+\left(\overset{´}{\kappa }-1\right)\left[-170\gamma +448\right]=448+\left(\overset{´}{\kappa }-1\right)\left[448-170\gamma \right]\\ & =\overset{´}{\kappa }\left[448-170\gamma \right]+170\gamma \end{array}$$

• **(b).** For $n=1\Rightarrow \overset{´}{\kappa }=2$ , $E_{2}=704$ , which is indeed true. Let $\overset{´}{\kappa }\geq 2$ , using Table 5, we get

1. **If** (⊤22)κ´−1→(⊤212)κ´ , then

GO2⊤212κ´=16β22⊤22κ´−1+2+30β23⊤22κ´−1+48β24⊤22κ´−1+2+84β34⊤22κ´−1+128β44⊤22κ´−1+1

GO2⊤212κ´=GO2⊤22κ´−1+256 (5.40)

2. **If** (⊤22)κ´−1→(⊤222)κ´ , then

GO2⊤222κ´=16β22⊤22κ´−1+2+30β23⊤22κ´−1+48β24⊤22κ´−1+2+84β34⊤22κ´−1+128β44⊤22κ´−1+1

GO2⊤222κ´=GO2⊤22κ´−1+256 (5.41)

3. **If** (⊤22)κ´−1→(⊤232)κ´ , then

GO2⊤232κ´=16β22⊤22κ´−1+2+30β23⊤22κ´−1+48β24⊤22κ´−1+2+84β34⊤22κ´−1+128β44⊤22κ´−1+1

GO2⊤232κ´=GO2⊤22κ´−1+256 (5.42)

Thus, we have

Eκ´GO2=γGO2⊤212κ´+γGO2⊤222κ´+1−2γGO2⊤232κ´

Using (6.40), (6.41) and (6.42), we get the following relation

Eκ´GO2=γGO2⊤22κ´−1+256+γGO2⊤22κ´−1+256+1−2γGO2⊤22κ´−1+256

Eκ´GO2=GO2⊤22κ´−1+256

$$E_{\overset{´}{\kappa }}^{GO_{2}}=E_{\overset{´}{\kappa }-1}^{GO_{2}}+256$$

Using recursive relation upto $\overset{´}{\kappa }-2$ terms

$$\begin{array}{cc} E_{\overset{´}{\kappa }}^{GO_{2}} & =E_{\overset{´}{\kappa }-\left(\overset{´}{\kappa }-2\right)}^{GO_{2}}+\left(\overset{´}{\kappa }-2\right)\left(256\right)=704+\left(\overset{´}{\kappa }-2\right)256\\ & =256\overset{´}{\kappa }+192 \end{array}$$

which completes the proof.

The expected values $E_{R_{1}}^{\overset{´}{\kappa }}$ , $E_{Re_{2}}^{\overset{´}{\kappa }}$ , $E_{HZ}^{\overset{´}{\kappa }}$ , $E_{ReZ_{2}}^{\overset{´}{\kappa }}$ , $E_{GO_{1}}^{\overset{´}{\kappa }}$ and $E_{GO_{2}}^{\overset{´}{\kappa }}$ descriptors for $\overset{´}{\kappa }=2n-1$ ; n∈N depend on γ , but the $E_{ReZ_{1}}^{\overset{´}{\kappa }}$ is independent of γ for both cases. As for the sake of generality we have taken expectations in odd and even cases. Therefore, at ${\left(\overset{´}{\kappa }-1\right)}^{th}$ stage the sum of expected values for $\overset{´}{\kappa }=2n-1$ and $\overset{´}{\kappa }=2n$ of a certain topological descriptor of three kink chains is equal to the sum of the average value of topological descriptor of three kink chain for $\overset{´}{\kappa }=2n-1$ and $\overset{´}{\kappa }=2n$ with a constant factor. As expectation of constant is zero, so our results are true. The values of $R_{1}$ , $R_{2}$ , HZ , $ReZ_{2}$ , $GO_{1}$ and $GO_{2}$ descriptors can be computed by using $\gamma =\frac{1}{3}$ in the above proved theorems.

Corollary 1 Let $\overset{´}{\kappa }=2n-1$ ; n∈N then at $\left(\overset{´}{\kappa }-1\right)$ stage;

$•\;R_{1}=\frac{100}{3}\left(\overset{´}{\kappa }-1\right)+82$

$•\;R_{2}=\frac{130}{3}\left(\overset{´}{\kappa }-1\right)+140$

$•\;ReZ_{2}=\frac{2867}{315}\left(\overset{´}{\kappa }-1\right)+\frac{1564}{105}$

$•\;HZ=\frac{752}{3}\left(\overset{´}{\kappa }-1\right)+334$

$•\;GO_{1}=98\left(\overset{´}{\kappa }-1\right)+142$

$•\;GO_{2}=\frac{1174}{3}\left(\overset{´}{\kappa }-1\right)+448$

Remark 1 The value of $ReZ_{1}$ descriptor at ${\left(\overset{´}{\kappa }-1\right)}^{th}$ stage for $\overset{´}{\kappa }=2n-1$ and $\overset{´}{\kappa }=2n$ ; n∈N is equal and independent of γ , i.e ;

ReZ1(⊤2p2)=4κ´+6 ; p=1 , 2 and 3

It is observed that the expected values $E_{R_{1}}^{\overset{´}{\kappa }}$ , $E_{ReZ_{1}}^{\overset{´}{\kappa }}$ , $E_{Re_{2}}^{\overset{´}{\kappa }}$ , $E_{HZ}^{\overset{´}{\kappa }}$ , $E_{ReZ_{2}}^{\overset{´}{\kappa }}$ , $E_{GO_{1}}^{\overset{´}{\kappa }}$ and $E_{GO_{2}}^{\overset{´}{\kappa }}$ for $\overset{´}{\kappa }=2n$ ; n∈N are independent of γ and depends only on $\overset{´}{\kappa }$ .

Remark 2 Let $\overset{´}{\kappa }=2n$ ,; n∈N then at $\left(\overset{´}{\kappa }-1\right)$ stage,

$•\;R_{1}=32\overset{´}{\kappa }+50$

$•\;R_{2}=52\overset{´}{\kappa }+88$

$•\;ReZ_{2}=\frac{20}{3}\overset{´}{\kappa }+\frac{288}{35}$

$•\;HZ=168\overset{´}{\kappa }+166$

$•\;GO_{1}=68\overset{´}{\kappa }+74$

$•\;GO_{2}=256\overset{´}{\kappa }+192$

### 5.2 Analytical expressions at ${\left(k-1\right)}^{th}$ stage

Now we analytically prove that at ${\left(\overset{´}{\kappa }-1\right)}^{th}$ stage, for any value of γ and $\overset{´}{\kappa }$ , the $2^{nd}$ *Gourava* descriptor is always greater than the remaining six descriptors, namely, ( $1^{st}$ and $2^{nd}$ ) *Revan* descriptors, ( $1^{st}$ and $2^{nd}$ ), *Zagreb* descriptors, *Hyper-Zagreb* descriptor and $1^{st}$ *Gourava* descriptor for $\overset{´}{\kappa }=2n-1$ and $\overset{´}{\kappa }=2n$ . All the expressions holds for $\gamma =\frac{1}{3}$ and for all $\overset{´}{\kappa }\in N$ .

Corollary 2 For $\overset{´}{\kappa }=2n-1$ and $\overset{´}{\kappa }=2n$ ; n∈N , we have

EGO2⊤2γ2κ´>EHZ⊤2γ2κ´

Proof. • **For** $\overset{´}{\kappa }=2n-1$

EGO2⊤2γ2κ´−EHZ⊤2γ2κ´=κ´448−170γ+170γ−κ´272−64γ+64γ+62=106γ1−κ´+2224κ´−31>0

which holds for $\gamma =\frac{1}{3}$ and for all $\overset{´}{\kappa }\in N$ , so we have

EGO2⊤2γ2κ´>EHZ⊤2γ2κ´

• **For** $\overset{´}{\kappa }=2n$

EGO2⊤2γ2κ´−EHZ⊤2γ2κ´=256κ´+192−168κ´+166=88κ´+26>0

which holds for all $\overset{´}{\kappa }\in N$ , so we have

EGO2⊤2γ2κ´>EHZ⊤2γ2κ´

Corollary 3 For $\overset{´}{\kappa }=2n-1$ and $\overset{´}{\kappa }=2n$ ; $\overset{´}{\kappa }\in N$ , we have

EHZ⊤2γ2κ´>EGO1⊤2γ2κ´

Proof. • **For** $\overset{´}{\kappa }=2n-1$

EHZ⊤2γ2κ´−EGO1⊤2γ2κ´=κ´272−64γ+64γ+62−κ´104−18γ+18γ+38=46γ1−κ´+2136κ´−19>0

which holds for $\gamma =\frac{1}{3}$ and for all $\overset{´}{\kappa }\in N$ , so we have

EHZ⊤2γ2κ´>EGO1⊤2γ2κ´

• For $\overset{´}{\kappa }=2n$

EHZ⊤2γ2κ´−EGO1⊤2γ2κ´=168κ´+166−68κ´+74=100κ´+92>0

which holds for all $\overset{´}{\kappa }\in N$ , so we have

EHZ⊤2γ2κ´>EGO1⊤2γ2κ´

Corollary 4 For $\overset{´}{\kappa }=2n-1$ and $\overset{´}{\kappa }=2n$ ; $\overset{´}{\kappa }\in N$ , we have

EGO1⊤2γ2κ´>ER2⊤2γ2κ´

Proof. • **For** $\overset{´}{\kappa }=2n-1$

EGO1⊤2γ2κ´−ER2⊤2γ2κ´=κ´104−18γ+18γ+38−κ´10γ+40−10γ+100=28γ1−κ´+232κ´−31>0

which holds for $\gamma =\frac{1}{3}$ and for all $\overset{´}{\kappa }\in N$ , so we have

EGO1⊤2γ2κ´>ER2⊤2γ2κ´

• **For** $\overset{´}{\kappa }=2n$

EGO1⊤2γ2κ´−ER2⊤2γ2κ´=68κ´+74−52κ´+88=16κ´−14>0

which holds for all $\overset{´}{\kappa }\in N$ , so we have

EGO1⊤2γ2κ´>ER2⊤2γ2κ´

Corollary 5 For $\overset{´}{\kappa }=2n-1$ and $\overset{´}{\kappa }=2n$ ; $\overset{´}{\kappa }\in N$ , we have

ER2⊤2γ2κ´>ER1⊤2γ2κ´

Proof. • **For** $\overset{´}{\kappa }=2n-1$

ER2⊤2γ2κ´−ER1⊤2γ2κ´=κ´10γ+40−10γ+100−κ´4γ+32−4γ+50=6γκ´−1+24κ´−25>0

which holds for $\gamma =\frac{1}{3}$ and for all $\overset{´}{\kappa }\in N$ , so we have

ER2⊤2γ2κ´>ER1⊤2γ2κ´

• For $\overset{´}{\kappa }=2n$

ER2⊤2γ2κ´−ER1⊤2γ2κ´=52κ´+88−32κ´+50=20κ´+38>0

which holds for all $\overset{´}{\kappa }\in N$ , so we have

ER2⊤2γ2κ´>ER1⊤2γ2κ´

Corollary 6 For $\overset{´}{\kappa }=2n-1$ and $\overset{´}{\kappa }=2n$ ; $\overset{´}{\kappa }\in N$ , we have

ER1⊤2γ2κ´>EReZ2⊤2γ2κ´

Proof. • **For** $\overset{´}{\kappa }=2n-1$

ER1⊤2γ2κ´−EReZ2⊤2γ2κ´=κ´4γ+32−4γ+50−κ´283−73105γ+584105+73105γ=493105γκ´−1+2334κ´−233335>0

which holds for $\gamma =\frac{1}{3}$ and for all $\overset{´}{\kappa }\in N$ , so we have

ER1⊤2γ2κ´>EReZ2⊤2γ2κ´

• For $\overset{´}{\kappa }=2n$

ER1⊤2γ2κ´−EReZ2⊤2γ2κ´=32κ´+50−203κ´+28835=763κ´+146235>0

which holds for all $\overset{´}{\kappa }\in N$ , so we have

ER1⊤2γ2κ´>EReZ2⊤2γ2κ´

Corollary 7 For $\overset{´}{\kappa }=2n-1$ and $\overset{´}{\kappa }=2n$ ; $\overset{´}{\kappa }\in N$ , we have

EReZ2⊤2γ2κ´>EReZ1⊤2γ2κ´

Proof. • **For** $\overset{´}{\kappa }=2n-1$

EReZ2⊤2γ2κ´−EReZ1⊤2γ2κ´=κ´283−73105γ+584105+73105γ−4κ´+6=73105γ1−κ´+238κ´−2335>0

which holds for $\gamma =\frac{1}{3}$ and for all $\overset{´}{\kappa }\in N$ , so we have

EReZ2⊤2γ2κ´>EReZ1⊤2γ2κ´

• For $\overset{´}{\kappa }=2n$

EReZ2⊤2γ2κ´−EReZ1⊤2γ2κ´=203κ´+28835−4κ´+6=83κ´+7835>0

which holds for all $\overset{´}{\kappa }\in N$ , so we have

EReZ2⊤2γ2κ´>EReZ1⊤2γ2κ´

From the above analytical expressions we get;

Corollary 8
EGO2⊤2γ2κ´>EHZ⊤2γ2κ´>EGO1⊤2γ2κ´>ER2⊤2γ2κ´>ER1⊤2γ2κ´>EReZ2⊤2γ2κ´>EReZ1⊤2γ2κ´

### 5.3 A comparison of expected values of topological descriptors at ${\left(\overset{´}{\kappa }-1\right)}^{th}$ stage

Table 6, 7 depict the expected values of *Revan* descriptors, *Zagreb* descriptors, *Hyper-Zagreb* descriptor and *Gourava* descriptors for $\gamma =\frac{1}{3}$ and $\overset{´}{\kappa }=2n-1$ and $\overset{´}{\kappa }=2n$ respectively. Observe that the value of expectation of $2^{nd}$ *Gourava* descriptor is always greater than the remaining six descriptors in both cases.

**TABLE 6:** Expected values for topological descriptors at ${\left(\overset{´}{\kappa }-1\right)}^{th}$ stage for $\gamma =\frac{1}{3}$ and $\overset{´}{\kappa }=2n-1$ .

$\overset{´}{\kappa }$	$E^{R_{1}}$	$E^{R_{2}}$	$E^{ReZ_{1}}$	$E^{ReZ_{2}}$	$E^{HZ}$	$E^{GO_{1}}$	$E^{GO_{2}}$
3	148.67	226.67	18	33.09	835.33	338	1,230.66
5	215.33	313.33	26	51.30	1,336.66	534	2013.33
7	282	400	34	69.50	1838	730	2,796
9	348.67	486.67	42	87.70	2,339.33	926	3,578.66
11	415.33	573.33	50	105.91	2,840.66	1,122	4,361.33
13	482	660	58	124.11	3,342	1,318	5,144
15	548.67	746.67	66	142.31	3,843.33	1,514	5,926.66
17	615.33	833.33	74	160.52	4,344.66	1710	6,709.33
19	682	920	82	178.72	4,864	1906	7,492
21	748.67	1,006.67	90	196.92	5,347.33	2,102	8,274.66

**TABLE 7:** Expected values for topological descriptors at ${\left(\overset{´}{\kappa }-1\right)}^{th}$ stage for $\gamma =\frac{1}{3}$ and $\overset{´}{\kappa }=2n$ .

$\overset{´}{\kappa }$	$E^{R_{1}}$	$E^{R_{2}}$	$E^{ReZ_{1}}$	$E^{ReZ_{2}}$	$E^{HZ}$	$E^{GO_{1}}$	$E^{GO_{2}}$
4	178	296	22	34.89	838	346	1,216
6	242	400	30	48.22	1,174	482	1728
8	306	504	38	61.56	1,510	618	2,240
10	370	608	46	74.89	1846	754	2,752
12	434	712	54	88.22	2,182	890	3,264
14	498	816	62	101.56	2,518	1,026	3,776
16	562	920	70	114.89	2,854	1,162	4,288
18	626	1,024	78	128.22	3,190	1,298	4,800
20	690	1,128	86	141.56	3,526	1,434	5,312
22	754	1,232	94	154.89	3,862	1,570	5,824

### 5.4 Graphical representation of expected values of topological descriptors at ${\left(\overset{´}{\kappa }-1\right)}^{th}$ stage

**FIGURE 13:**
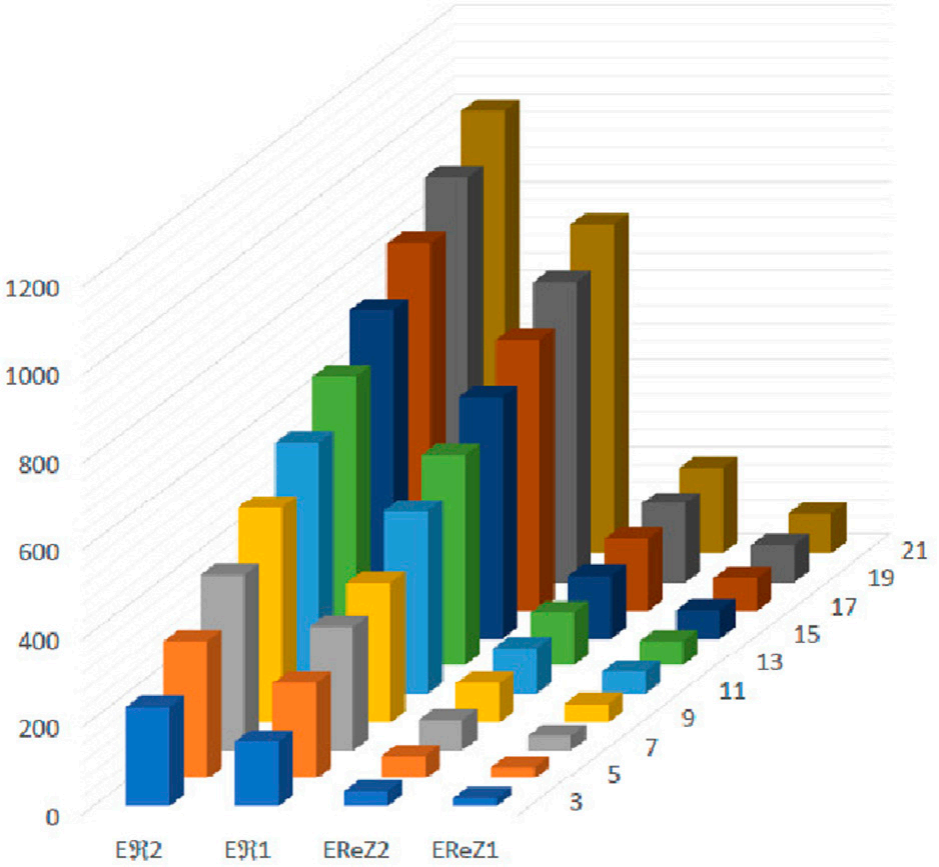
Graphical representation of expectations of $R_{1}$ , $R_{2}$ , $ReZ_{1}$ and $ReZ_{2}$ at ${\left(\overset{´}{\kappa }-1\right)}^{th}$ stage for $\overset{´}{\kappa }=2n-1$ .

**FIGURE 14:**
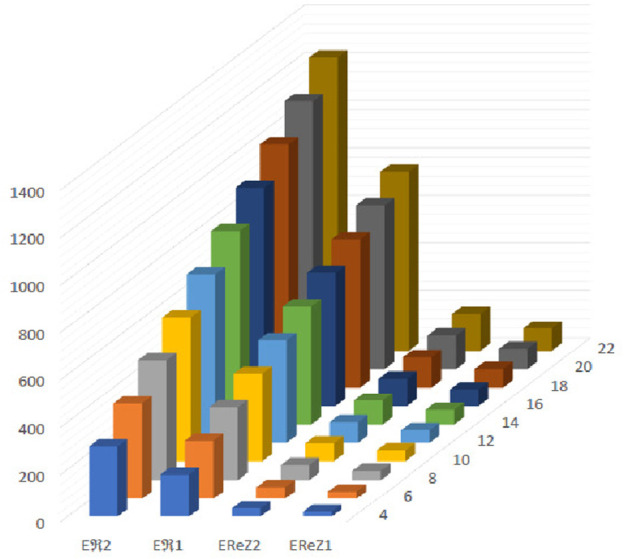
Graphical representation of expectations of $R_{1}$ , $R_{2}$ , $ReZ_{1}$ and $ReZ_{2}$ at ${\left(\overset{´}{\kappa }-1\right)}^{th}$ stage for $\overset{´}{\kappa }=2n$ .

**FIGURE 15:**
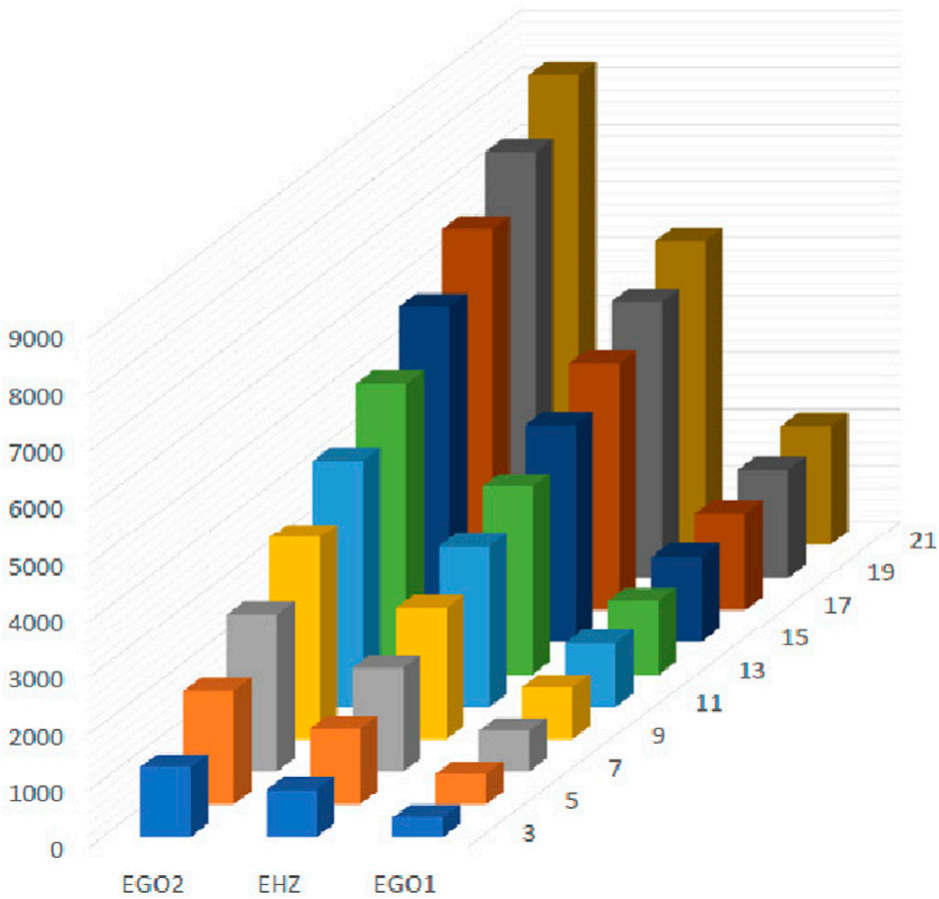
Graphical representation of expectations of HZ , $GO_{1}$ and $GO_{2}$ at ${\left(\overset{´}{\kappa }-1\right)}^{th}$ stage for $\overset{´}{\kappa }=2n-1$ .

**FIGURE 16:**
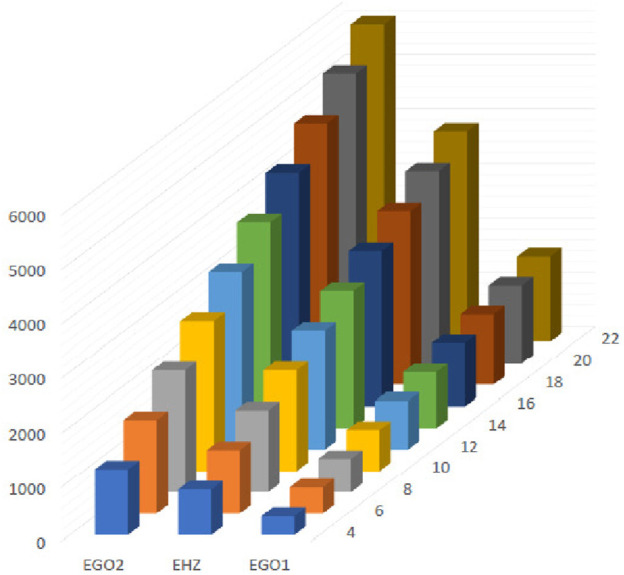
Graphical representation of expectations of HZ , $GO_{1}$ and $GO_{2}$ at ${\left(\overset{´}{\kappa }-1\right)}^{th}$ stage for $\overset{´}{\kappa }=2n$ .

The Figures 13–16 shows that expectation of $2^{nd}$ *Gourava* descriptor attains maximum value and of $1^{st}$ redefined *Zagreb* descriptor attains minimum value at ${\left(\overset{´}{\kappa }-1\right)}^{th}$ stage for both the cases.

## 6 Applications

Kinks, which denote abrupt changes in the direction of edges within a graph, hold notable applications across diverse fields. In circuit design, minimizing kinks optimizes wire lengths and enhances efficiency. Network routing benefits from understanding kinks, as they affect data flow and network performance. Transportation planners use kink analysis to streamline traffic, plan intersections, and design efficient road networks. Graph drawing algorithms consider kinks for aesthetically pleasing and comprehensible visual representations. Lastly, in various applications where visual appeal matters, reducing kinks enhances the attractiveness and clarity of graph representations.

Studying the interforce interactions and scattering of (Lima and Almeida, 2023) kink-antikink-like solutions in a two-dimensional dilaton gravity model has practical implications in fields like material science, nonlinear optics, and cosmology. It aids in understanding energy distribution, stability dynamics, and defect interactions, which are crucial for developing advanced technologies and predicting behaviors in complex physical systems. The *in situ* (Zhu et al., 2023) investigations have revealed the important role for the kinks. During the growth, the creation of kinks determines the growth rate. Besides, when two domains coalesce, the shape of the final flake is affected by kinks.

In computer graphics, square and hexagonal grids are frequently used to create images or simulations. Kinks in these grids can represent corners or junctions in a digital image. Hexagonal kinks are essential in the study of tessellation and pattern generation. The expected value of random graphs plays a crucial role in graph theory as well. It helps analyze and predict various graph properties in probabilistic settings. By studying expected values in graph theory, we gain a balanced understanding of how certain structures behave under randomness, which informs both theory and practical applications. Expected values are used to calculate the probability that a random graph is connected. It can be employed to estimate the likelihood that two randomly generated graphs are isomorphic. This is valuable in assessing the structural similarity between graphs.

## 7 Conclusion

In this research work, we determined $R_{1}$ , $R_{2}$ , $ReZ_{1}$ , $ReZ_{2}$ , HZ , $GO_{1}$ and $GO_{2}$ descriptors for the graphical structures of kink chains of type ⊤22 named as ⊤212 , ⊤222 and ⊤232 . We infered that $GO_{2}$ is a maximizing, while $ReZ_{1}$ is a minimizing descriptor of ⊤212 , ⊤222 and ⊤232 ,for both, odd and even case. Further we determined expected values of topological descriptors of (⊤2γ2)k at ${\left(\overset{´}{\kappa }-1\right)}^{th}$ stage. We analyzed that value of $ReZ_{1}$ descriptor of ⊤212 , ⊤222 and ⊤232 and $E_{k}^{ReZ_{1}}$ is same and independent of γ at ${\left(\overset{´}{\kappa }-1\right)}^{th}$ stage. We made numerical comparison for these expected values at ${\left(\overset{´}{\kappa }-1\right)}^{th}$ stage and conclude that expected value, $E_{k}^{GO_{2}}$ is greater while expected value, $E_{k}^{ReZ_{1}}$ is smaller among other expectations $E_{k}^{R_{1}}$ , $E_{k}^{R_{2}}$ , and $E_{k}^{ReZ_{2}}$ , $E_{k}^{HZ}$ , and $E_{k}^{GO_{1}}$ at ${\left(\overset{´}{\kappa }-1\right)}^{th}$ stage. Also, we gave exact analytical expressions of this comparison at ${\left(\overset{´}{\kappa }-1\right)}^{th}$ stage which agree with numerical values of comparisons. Results at ${\left(\overset{´}{\kappa }-2\right)}^{th}$ stage will be computed in the next article.

## Data Availability

The original contributions presented in the study are included in the article/supplementary material, further inquiries can be directed to the corresponding authors.
